# High dose expression of heme oxigenase-1 induces retinal degeneration through ER stress-related DDIT3

**DOI:** 10.1186/s13024-021-00437-4

**Published:** 2021-03-10

**Authors:** Huirong Li, Bo Liu, Lili Lian, Jiajia Zhou, Shengjin Xiang, Yifan Zhai, Yu Chen, Xiaoyin Ma, Wencan Wu, Ling Hou

**Affiliations:** 1 Laboratory of Developmental Cell Biology and Disease, School of Ophthalmology and Optometry and Eye Hospital, and State Key Laboratory of Ophthalmology, Optometry and Vision Science, Wenzhou Medical University, Wenzhou, China; 2 Eye Hospital of Wenzhou Medical University, Wenzhou, 325003 China

**Keywords:** Photoreceptor degeneration, Antioxidant enzyme, Recombinant adeno-associated virus, Endoplasmic reticulum stress, CHOP pathway

## Abstract

**Background:**

Oxidative stress is a common cause of neurodegeneration and plays a central role in retinal degenerative diseases. Heme oxygenase-1 (HMOX1) is a redox-regulated enzyme that is induced in neurodegenerative diseases and acts against oxidative stress but can also promote cell death, a phenomenon that is still unexplained in molecular terms. Here, we test whether HMOX1 has opposing effects during retinal degeneration and investigate the molecular mechanisms behind its pro-apoptotic role.

**Methods:**

Basal and induced levels of HMOX1 in retinas are examined during light-induced retinal degeneration in mice. Light damage-independent HMOX1 induction at two different expression levels is achieved by intraocular injection of different doses of an adeno-associated virus vector expressing HMOX1. Activation of Müller glial cells, retinal morphology and photoreceptor cell death are examined using hematoxylin-eosin staining, TUNEL assays, immunostaining and retinal function are evaluated with electroretinograms. Downstream gene expression of HMOX1 is analyzed by RNA-seq, qPCR examination and western blotting. The role of one of these genes, the pro-apoptotic DNA damage inducible transcript 3 (*Ddit3*), is analyzed in a line of knockout mice.

**Results:**

Light-induced retinal degeneration leads to photoreceptor degeneration and concomitant HMOX1 induction. HMOX1 expression at low levels before light exposure prevents photoreceptor degeneration but expression at high levels directly induces photoreceptor degeneration even without light stress. Photoreceptor degeneration following high level expression of HMOX1 is associated with a mislocalization of rhodopsin in photoreceptors and an increase in the expression of DDIT3. Genetic deletion of *Ddit3* in knockout mice prevents photoreceptor cell degeneration normally resulting from high level HMOX1 expression.

**Conclusion:**

The results reveal that the expression levels determine whether HMOX1 is protective or deleterious in the retina. Furthermore, in contrast to the protective low dose of HMOX1, the deleterious high dose is associated with induction of DDIT3 and endoplasmic reticulum stress as manifested, for instance, in rhodopsin mislocalization. Hence, future applications of *HMOX1* or its regulated targets in gene therapy approaches should carefully consider expression levels in order to avoid potentially devastating effects.

**Supplementary Information:**

The online version contains supplementary material available at 10.1186/s13024-021-00437-4.

## Background

Neurodegenerative diseases are a major threat to human health and welfare in the world. Oxidative injury is a common feature of neurodegenerative diseases such as Alzheimer’s disease (AD) [1] and Parkinson’s disease (PD) [2] and is one of the main contributing factors involved in photoreceptor cell loss seen in retinal degenerative diseases such as retinitis pigmentosa (RP) and age-related macular degeneration (AMD), the leading cause of profound and permanent vision loss in developed countries and highly common among older people [3–6]. Therefore, general antioxidant treatment may be an appropriate approach to prevent retinal degeneration, but serious side effects have so far hindered success in clinical trials. It is paramount, therefore, to explore in more detail the oxidative stress pathway during retinal degeneration in order to identify more specific therapeutic targets.

Heme oxygenase-1 (Hmox1) is a sensor of oxidative stress that is induced as part of the general response to oxidative stress in a variety of mammalian cells [7]. HMOX1 is capable of reducing oxidative stress because of the consumption of molecular oxygen in the heme oxygenase reaction pathway where it catalyzes the degradation of heme b (iron protoporphyrin IX) to carbon monoxide (CO), ferrous iron and biliverdin IX [8]. In addition, its catalyzed metabolites, such as CO and biliverdin, are able to exert strong antioxidant, anti-apoptotic and anti-inflammatory activities [9, 10]. In both human and mouse, germline genetic deficiencies of *Hmox1* render multiple tissues vulnerable to oxidative stress [11–13] and experimental overexpression of *Hmox1* in mouse astrocytes attenuates neuronal cell death caused by oxidative stress [14]. Therefore, HMOX1 induction is recognized as an endogenous protective system to prevent oxidative injury in the nervous system.

Induction of HMOX1, however, has also been associated with neuronal damage and degeneration [9, 15–17]. For instance, accumulation of *HMOX1* in astroglial cells seems to be a core signature of the aging and diseased CNS and may be involved in CNS pathology [15] such as in the above mentioned AD [16, 17] and PD [17–19]. In addition, HMOX1 overactivation triggers cell death in glioma cells [20]. However, the conditions that decide whether HMOX1 plays a protective or a deleterious role are still unclear.

Recently, it has been shown that the deleterious effect of HMOX1 induction may be associated with its catalyzed metabolite ferrous iron, which accelerates mitochondrial iron sequestration [15, 21]. Moreover, as HMOX1 is an endoplasmic reticulum (ER)-anchored protein [7], it is conceivable that it plays a role in ER stress. The ER is the central intracellular organelle where protein folding, transport and post-translational modifications take place to allow translocation of transmembrane proteins to the Golgi apparatus and ultimately to vesicles for secretion or display on the plasma surface [22]. An abundance of unfolded or misfolded proteins in the ER leads to accumulation of these proteins in the ER lumen and in turn to the unfolded protein response (UPR), a sign of ER stress [22]. When ER stress is severe and prolonged, the UPR can lead to an upregulated expression of the DNA damage inducible transcript 3 (DDIT3, also known as transcription factor C/EBP-homologous protein, CHOP). DDIT3 is an ER stress effector that promotes cell death [23, 24]. Not surprisingly; ER stress is involved in neurodegeneration [22, 25], but there is yet another mechanism by which HMOX1 might exert its deleterious effects. The protein can be truncated into a smaller peptide that is abnormally translocated from the ER to mitochondria, in turn leading to mitochondrial dysfunction and oxidative stress [15, 26–28]. Interestingly, ER stress can also lead to induction of HMOX1 [29], but the molecular mechanisms of the involvement of HMOX1 in ER homeostasis still needs to be worked out.

HMOX1 induction has been observed during retinal damage or degeneration, and overexpression of HMOX1 by AAV-mediated gene delivery can partially prevent photoreceptor degeneration [30, 31] and its activity is required for preventing retinal degeneration in a mouse model of RP [32]. Nevertheless, the above observations that HMOX1 can be deleterious to the CNS and that the neural retina is part of the CNS, prompted us to explore in more detail the role of HMOX1 in retinal homeostasis in a model of light-induced retinal degeneration in mice. These studies revealed that under light damage, HMOX1 is induced and is associated with an aggravation of retinal degeneration. Nevertheless, expression of a low dose of recombinant HMOX1 using an AAV8 vector protects against subsequent light-induced photoreceptor cell degeneration, but expression of a high dose of HMOX1 causes photoreceptor cell death and retinal degeneration. In fact, high level accumulation of HMOX1 leads to ER stress, an increase in the expression of the above mentioned *Ddit3,* and aberrant localization of rhodopsin in the photoreceptor cell body instead of the outer segment. Importantly, genetic deletion of *Ddit3* blocks this rhodopsin mislocalization and prevents photoreceptor cell death caused by high level expression of HMOX1.

## Materials and methods

### Animals and Hematoxylin-Eosin staining

Two-month-old BALB/c mice (*Tyr*^*c*^*/Tyr*^*c*^) were purchased from Vital River Laboratory in Beijing and C57BL6/J mice were obtained from Charles River Laboratories in Shanghai, China. *Ddit3−/−* mice on a C57BL/6 J background were purchased from Jackson laboratory (Stock number: 005530), and .the primers (F: ATG CCC TTA CCT ATC GTG, R1: AAC GCC AGG GTT TTC CCA GTC A, R2: GCA GGG TCA AGA GTA GTG) were used for genotyping *Ddit3* mutant alle (F and R1) or wildtype alle (F and R2). All mice were fed a standard laboratory chow and maintained in a temperature-controlled room at 21–23 °C with a 12 h-light/12 h-dark photoperiod. All animal experiments were carried out in accordance with the approved guidelines of the Wenzhou Medical University Institutional Animal Care and Use Committee (Permit Number: WZMCOPT- 090316).

Hematoxylin-Eosin staining has been described previously [30]. The thickness of photoreceptor layer (PL), included outer nuclear layer (ONL), inner segment layer (IS) and outer segment layer (OS), was measured at a distance between 100 μm and 1200 μm from the optic nerve head to the peripheral retina.

### Light damage

The procedure to induce light damage has been reported [30]. Briefly, two-month-old albino mice were constantly exposed to LED light (15,000 lx) in non-refractive cages for the indicated days (1, 3 or 5 days), then were immediately subjected to retinal degeneration analysis. During light exposure, mice were kept at 1 or 2 per cage and were allowed free access to food and water.

### Quantitative real-time PCR

Total neural retinal RNAs were extracted by Trizol reagent (Invitrogen) and converted to cDNA using a reverse transcriptase kit (Promega). Quantitative real-time PCR was performed on a 7500 Real-Time PCR Detection System (Applied Biosystems) with Power SYBR Green PCR Master Mix as previously report [33]. All primer sequences used for real-time PCR are listed in the additional file, Table S1.

### Immunostaining and TUNEL analysis

For immunostaining, eye cups were dissected in PBS, fixed in 4% PFA for 2 h, dehydrated in 30% sucrose at 4 °C overnight and then frozen in OCT compound. Cryostat sections (12 μm) were blocked using 3% BSA at room temperature (RT) for 30 min. Primary antibodies were: anti-HMOX1 (1:50; Santa Cruz Biotechnology, sc-10,789), anti-Rhodopsin (1:200; Millipore, MAB5316), anti-GFAP (1:300; Abcam, ab7260), anti-Opsin (1:200; Millipore, AB5745), anti-DDIT3 (1:200; Beyotime, AC532), anti-Recoverin (1:200; Millipore, AB5585) or anti-EGFP (1:300; Abcam, ab1218). The primary antibodies were revealed with Alexa 594-conjugated or Alexa 488-conjugated donkey anti-mouse or anti-rabbit at RT for 1 h.

TUNEL staining was done using the TUNEL Kit #11684795910 from Roche according to the manufacturer’s protocol.

### Cell culture and treatment

The 661 W photoreceptor cell line was generously provided by Dr. Muayyad Al-Ubaidi and the cells were routinely cultured in Dulbecco’s modified Eagle’s medium (DMEM, Gibco) supplemented with 10% FBS and 1% penicillin/streptomycin and kept at 37 °C in a humidified atmosphere with 5% CO_2_. The cells (1 × 10^5^) were seeded in 6-well plates and grown for 24 h prior to ferric ammonium citrate (FAC) treatments. The different doses of FAC (0, 10, 25, 50, 100, 200, 400 μM) were used to stimulate 661 W cells for 2 days before western blotting analysis.

### Western blotting

Neural retinas were isolated from mice and added to 1.5 ml centrifuge tubes containing 100 μl SDS lysis solution (Byotime, China) and protease inhibitors (Cocktail Set I, Calbiochem). For cell protein extraction, 661 W cells were washed with PBS for three times and lysed with SDS lysis solution on ice for 30 min. Western blotting was performed as described previously [30]. Primary antibodies were: anti-HMOX1 (1:500; Santa Cruz Biotechnology, sc-10789), anti-α-Tubulin (1:1000; Santa Cruz Biotechnology, sc-53646), anti-GAPDH (1:2000; KANGCHEN Excellence for Research, KC-5G4), anti-DDIT3 (1:1000; Beyotime, AC532), anti-ATF4 (1:1000, Novus, H00000468-MO1) or anti-EGFP (1:1000; Abcam,ab1218). Donkey anti-mouse IgG (H + L) IRDye® 800CW (LI-COR, C70502-02) or donkey anti-rabbit IgG (H + L) IRDye® 800CW (LI-COR, C70502-02) were used as secondary antibodies. The protein bands were scanned using a LI-COR machine. Mouse anti-α-Tubulin or GAPDH antibodies were used as internal controls to normalize for the amount of total proteins.

### AAV8 vector construction and virus injection

Adeno-associated virus 8 (AAV8)-mediated expression of HMOX1 (hereafter called AAV8-HMOX1) was generated as previously reported [30]. For the high dose of AAV8-HMOX1 (2 × 10^9^ genome copies), adult mice were intravitreally injected with 1 μl of AAV8-HMOX1 virus (2 × 10^12^ genome copies/ml), using a pulled angled glass pipette under direct observation aided by a dissecting microscope under dim light. For the low dose of AAV8-HMOX1, the stock of AAV8-HMOX1 virus was diluted four times by the diluent (5% Glycerol in 1 × PBS), and 1 μl of the diluted virus (5 × 10^8^ genome copies) was injected into the intraocular space of the mice. Following injections, 1% atropine eye drops, tetracycline, and cortisone acetate eye ointments were applied. AAV8-GFP virus was purchased from Genechem (Shanghai, China) and injected into the intraocular space as described above for AAV8-HMOX1.

### Non-heme iron examination

Examination of iron levels was performed as previously reported, with small modifications [34]. Briefly, neural retinas were dissected in PBS, and each whole neural retina was incubated with 50 μl of 10% trichloroacetic acid (Sigma) and 3 M *hydrochloric acid* and subjected to shaking for 20 h at 1200 rpm at 65 °C. Then, the samples were mixed with the reaction buffer (10% thioglycolic acid in 1 M sodium acetate solution) followed by a mixture of the colorimetric solution (1% bathophenanthroline disulfonic acid) in a 1:1 volume ratio. The optical density was measured at 535 nm using endpoint examination in a microplate reader (SpectraMax M5). To quantify the relative level of non-heme iron, the value of light absorption from the control neural retinas without virus infection served as benchmark, and the fold change of iron level was calculated from relative ratio among the three groups.

### Electroretinography

Electroretinography (ERG) was performed as previously described [35]. Briefly, mice were dark-adapted overnight and anesthetized with a mixture of ketamine and xylazine. After 5 min of dilation, mice were stimulated by flash light varying in intensity from − 5.0 to 35 log scotopic candlepower-sec/m^2^ (cd/m^2^) in a Ganzfeld dome (Roland Q400, Wiesbaden, Germany). For light-adapted ERG recordings, a background light of 30 cd/m^2^ was applied to suppress rod responses. The stimulus light intensity was attenuated with neutral density filters (Kodak, Rochester, NY) and luminance was calibrated with an IL-1700 integrating radiometer/photometer (International Light, Newburyport, MA).

### Statistical analysis

Each experiment was repeated at least three times and results are presented as mean ± standard deviation (SD). Statistical analyses were performed using GraphPad Prism 8 with Student’s *T*-test when comparing two groups, and one-way ANOVA with Bonferroni post-hoc test was used for statistical analyses when comparing more than two groups. *p* value < 0.05 was considered significant.

## Results

### Induction of HMOX1 is associated with aggravation of retinal degeneration under light damage

**Fig. 1:**
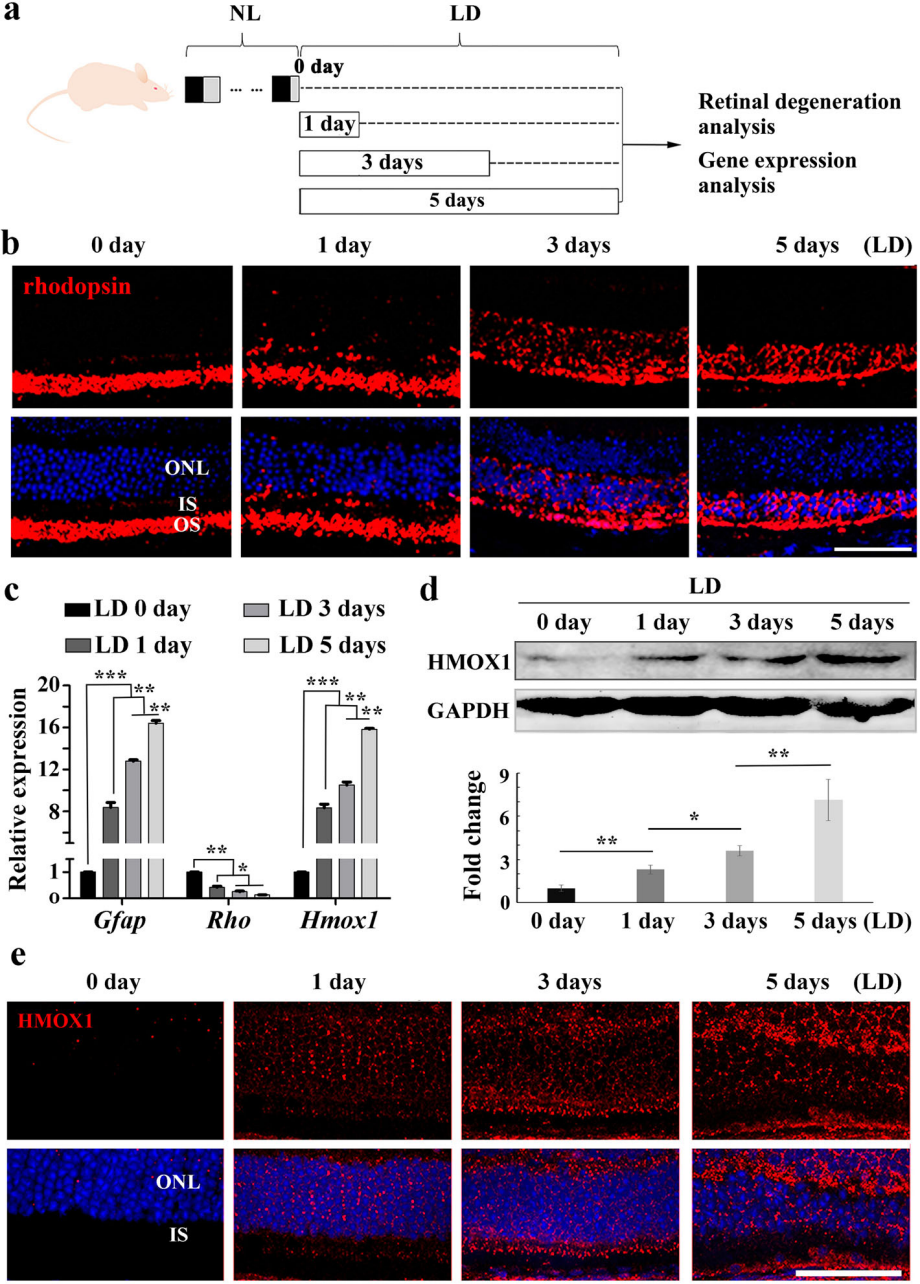
The level of HMOX1 induction depends on the extent of retinal degeneration. **a** Schematic representation of time frame and analysis of light damage (LD). Albino mice were raised in normal light (NL) for 2 months and then exposed to constant white light of 15,000 lx (LD) for 0, 1, 3 or 5 days to induce retinal damage. **b** Rhodopsin immunostaining of retinas from 2-month-old albino mice kept under high intensity light (15,000 lx) for 0, 1, 3 or 5 days. **c** Quantification of PCR show the expression levels of *Gfap*, *Rho* and *Hmox1* in the retinas under the indicated conditions (Error bars: SD; *n* = 3, one-way ANOVA). **d** Western blots of anti-HMOX1 and anti-GAPDH in the neural retinas of albino mice after light damage (upper panels) and bar graphs showing quantification of HMOX1 expression according to results of the above blots (lower panel) (Error bars: SD; *n* = 3, one-way ANOVA). **e** Anti-HMOX1 immunostaining of frozen sections of retinas from albino mice under the indicated conditions. ONL, outer nuclear layer; IS, photoreceptor inner segments; OS, photoreceptor outer segments. * or ** or *** indicates *p* < 0.05 or *p* < 0.01 or *p* < 0.001. Scale bars: 50 μm

Excessive light is the most obvious factor that induces HMOX1 expression in neural retinas and renders photoreceptor cells vulnerable to degeneration [36–38]. Hence, to investigate the relationship of retinal degeneration and induction of HMOX1, we exposed light-sensitive two-month-old BALB/c albino mice to light at 15,000 lx for different times (Fig. 1a). Immunohistochemistry showed that rhodopsin was normally localized in the outer segment layer of the retina but with increasing duration of light exposure was gradually mislocalized to the outer nuclear layer (ONL) (Fig. 1b). In addition, the photoreceptor layer (PL) became thinner (Additional file 1: Figure S1a and b), glial fibrillary acidic protein (GFAP) was significantly upregulated in Müller glial cells, and the rate of cell death rapidly increased in the ONL (Additional file 1: Figure S1c-e). These data indicate that photoreceptor degeneration became more severe with increasing time of light exposure. Moreover, Q-RT-PCR and WB data consistently showed that *Hmox1* RNA expression was upregulated in retinas after LD and that the levels of HMOX1 protein increased with the duration of light exposure (Fig. 1c and d). IF data also showed that HMOX1 expression was significantly increased in photoreceptor cells (Fig. 1e). These results indicate that the expression of HMOX1 is increased with increased photoreceptor degeneration and suggest that high levels of HMOX1 may be harmful to photoreceptor cells.

### Excessive HMOX1 leads to photoreceptor degeneration

**Fig. 2:**
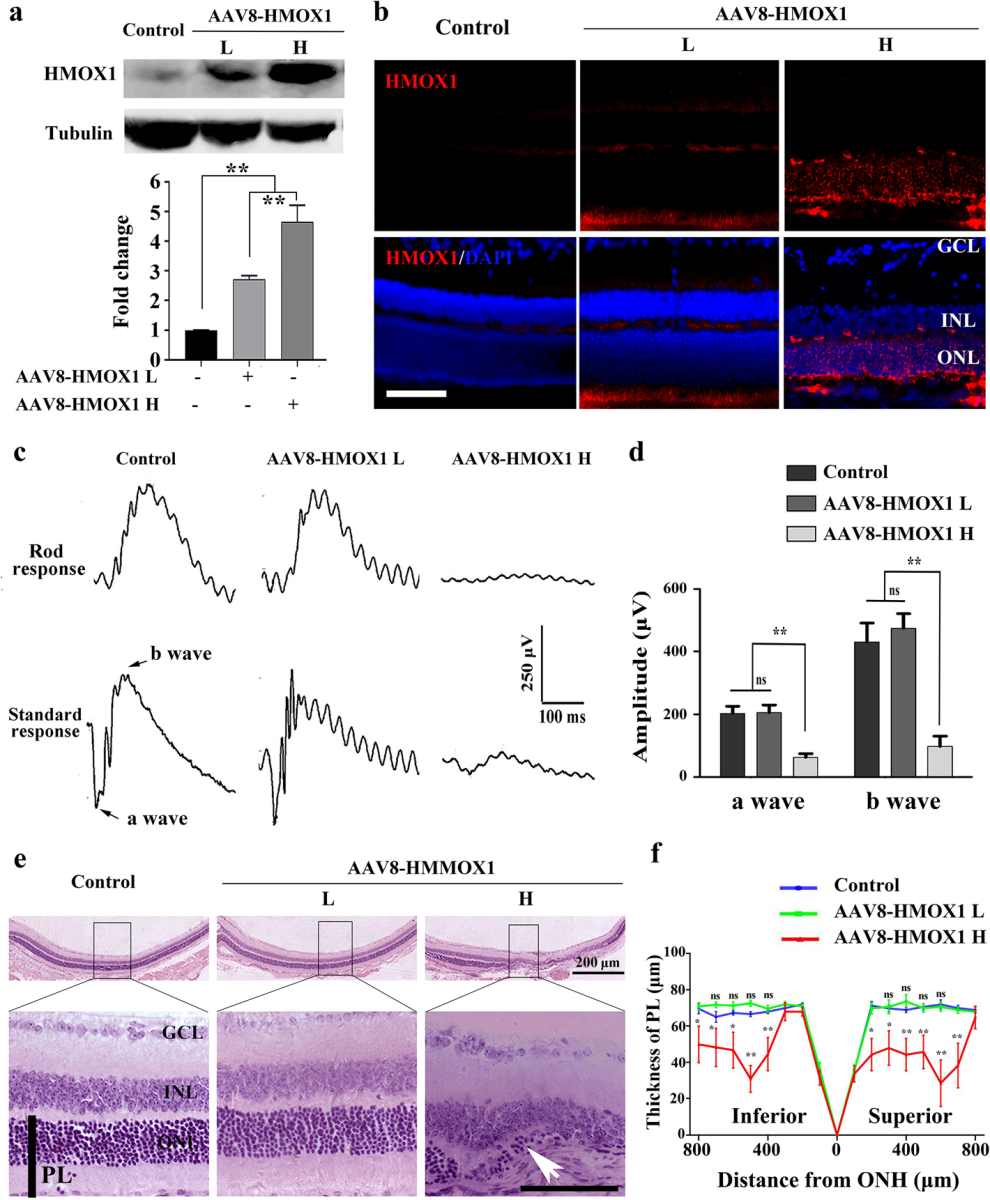
Overexpression of HMOX1 following high dose AAV8-HMOX1 infection disrupts retinal function and leads to retinal degeneration**. a** Western blots (upper panels) show the expression of HMOX1 in retinas from albino mice 2 weeks after infection with a low (5 × 10^8^ genome copies/eye) or a high dose (2 × 10^9^ genome copies /eye) of AAV-HMOX1 virus and bar graphs showing quantification of HMOX1 expression according to results of the immunoblots (Error bars: SD; *n* = 3, one-way ANOVA). **b** Anti-HMOX1 immunostaining of frozen sections of retinas from albino mice 2 weeks after infection. **c** ERG scotopic traces of rod response (upper panels) and standard response (lower panels) of albino mice under the indicated conditions. **d** Quantification of a- and b-wave amplitudes according to results of the above ERG scotopic traces (Error bars: SD; *n* = 6, one-way ANOVA). **e** Histological structure of retinas from albino mice 2 weeks after infection with low or high dose of AAV-HMOX1 virus. **f** Quantification of the thickness of the photoreceptor cell layer (including outer nuclear layer, inner segment layer and outer segment layer) from mice under the indicated conditions (Error bars: SD; *n* = 6, one-way ANOVA). AAV-HMOX1 L, low dose of AAV-HMOX1; AAV-HMOX1 H, high dose of AAV-HMOX1; ONL: outer nuclear layer; IS: photoreceptor inner segments; OS: photoreceptor outer segments; ns indicates no significant difference. * or ** indicates *p* < 0.05 or *p* < 0.01. Scale bars: 50 μm

Given that *Hmox1* induction was correlated with aggravation of photoreceptor degeneration, we explored whether excessive HMOX1 levels are deleterious to photoreceptor cells. To this end, we expressed different levels of HMOX1 using two different doses of a recombinant adeno-associated virus 8 (AAV8) (5 × 10^8^ versus 2 × 10^9^ viral genomes per eye). Two weeks after infection, WB data confirmed that injection of the lower dose increased expression of Hmox1 by 2.17 ± 0.13 fold (*n* = 3) compared to control, while injection of the higher dose increased it by 4.66 ± 0.56 fold, *n* = 3 (Fig. 2a). Consistently, IF data also showed that virus infection led to a dose-dependent increase in expression of HMOX1 in photoreceptor cells (Fig. 2b). We then tested the retinal function of the infected eyes by ERG examination. The data showed that the a-wave and b-wave amplitudes were dramatically reduced by the injection of the high dose of AAV8-HMOX1 (62.82 ± 29.18 μV and 97.25 ± 81.47 μV, respectively, *n* = 6), while the injection of the low dose did not affect the ERG amplitudes (a wave amplitude: 205.7 ± 59.72 μV; b wave amplitude: 474.1 ± 116.1 μV; *n* = 6, versus control a wave amplitude: 203.1 ± 56.77 μV; b wave amplitude: 430.3 ± 149.5 μV; *n* = 6) (Fig. 2c and d). Furthermore, histological analysis showed that the low dose did not cause any detectable change in retinal structure and function, but that the high dose led to a significant decrease of PL thickness, especially in the middle regions of both the superior and inferior retinas from ONH to periphery (Fig. 2e and f). These results indicate that overexpression of HMOX1 driven by a high dose of AAV8-HMOX1 disrupts retinal structure and function and leads to retinal degeneration.

**Fig. 3:**
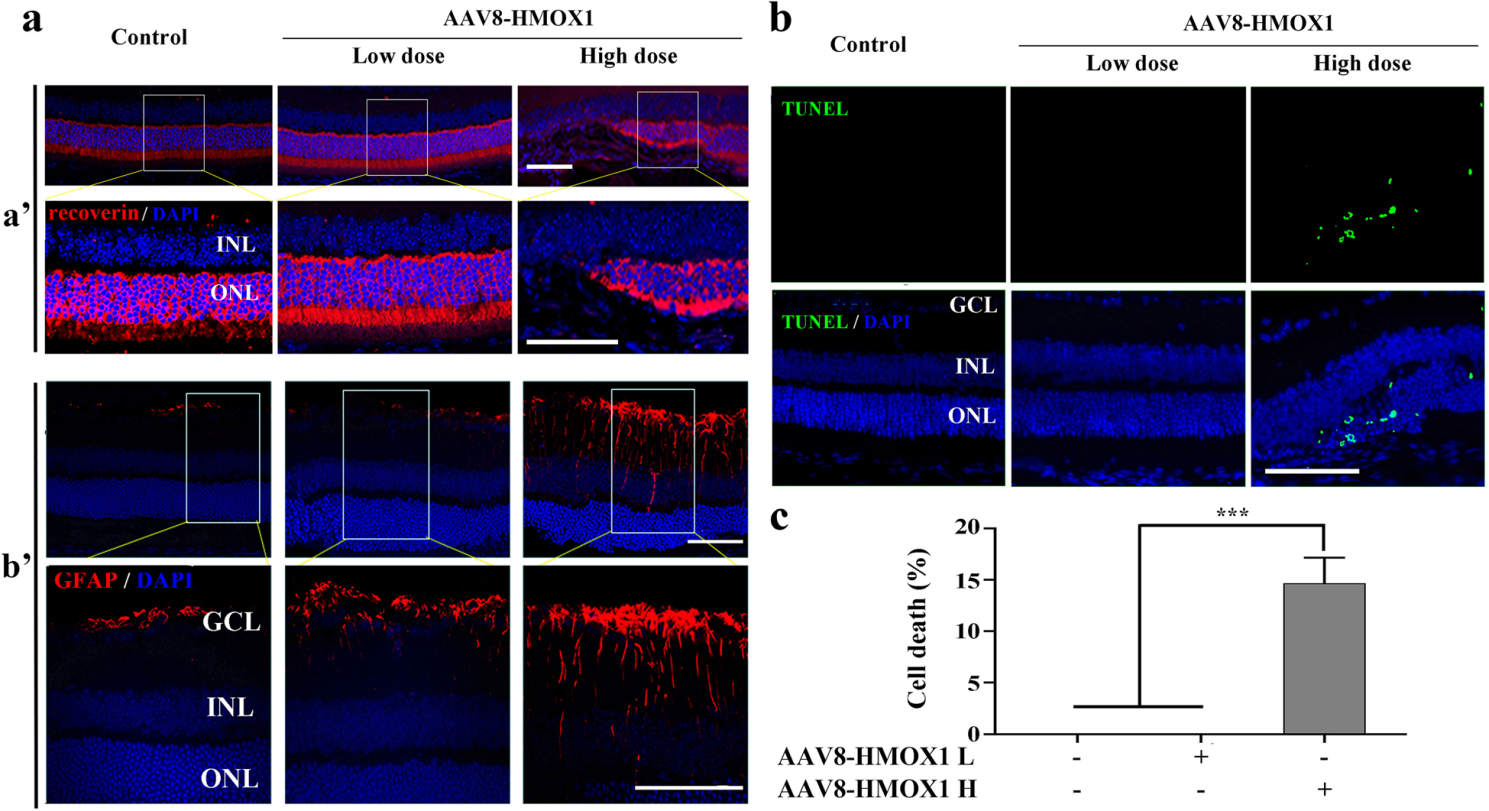
High dose of AAV-HMOX1 causes photoreceptor degeneration. **a** Immunostaining of Recoverin (panels a') and GFAP (panels b') in the retinas from albino mice 2 weeks after infection with a low or a high dose of AAV8-HMOX1 virus. **b, c** Cell death analysis by TUNEL assay in retinas from albino mice under the indicated conditions **b** and the quantification of the TUNEL-positive cells in the photoreceptor cell layer of the mice **c** (Error bars: SD; *n* = 3, Student *T*-test). AAV-HMOX1 L, low dose of AAV-HMOX1; AAV-HMOX1 H, high dose of AAV-HMOX1; GCL:ganglion cell layer; INL:inner nuclear layer; ONL: outer nuclear layer; IS: photoreceptor inner segments; OS: photoreceptor outer segments; ns, no significant difference; *** indicates *p* < 0.001. Scale bars: 50 μm

To further confirm photoreceptor degeneration, we examined the expression of recoverin (a photoreceptor cell marker) by immunostaining of cryostat sections. As shown in Fig. 3, compared with the control group, the signal of recoverin immunoreactivity almost disappeared in the middle region of the neural retinas after high dose infection with AAV8-HMOX1 but remained unchanged after low dose infection (Fig. 3a, panels a’). Furthermore, in high dose- but not low dose-infected neural retinas, GFAP expression was induced in Müller glial cells (Fig. 3a, panels b’), along with the appearance of TUNEL positive signals in photoreceptor cells [14.67 ± 2.52% (TUNEL rate of ONL), *n* = 3] (Fig. 3b and c). Hence, high dose infection leads to severe photoreceptor degeneration, in contrast to low dose infection, which prevented light-induced photoreceptor degeneration (Additional file 2: Figure S2) as previously reported [30, 31].

**Fig. 4:**
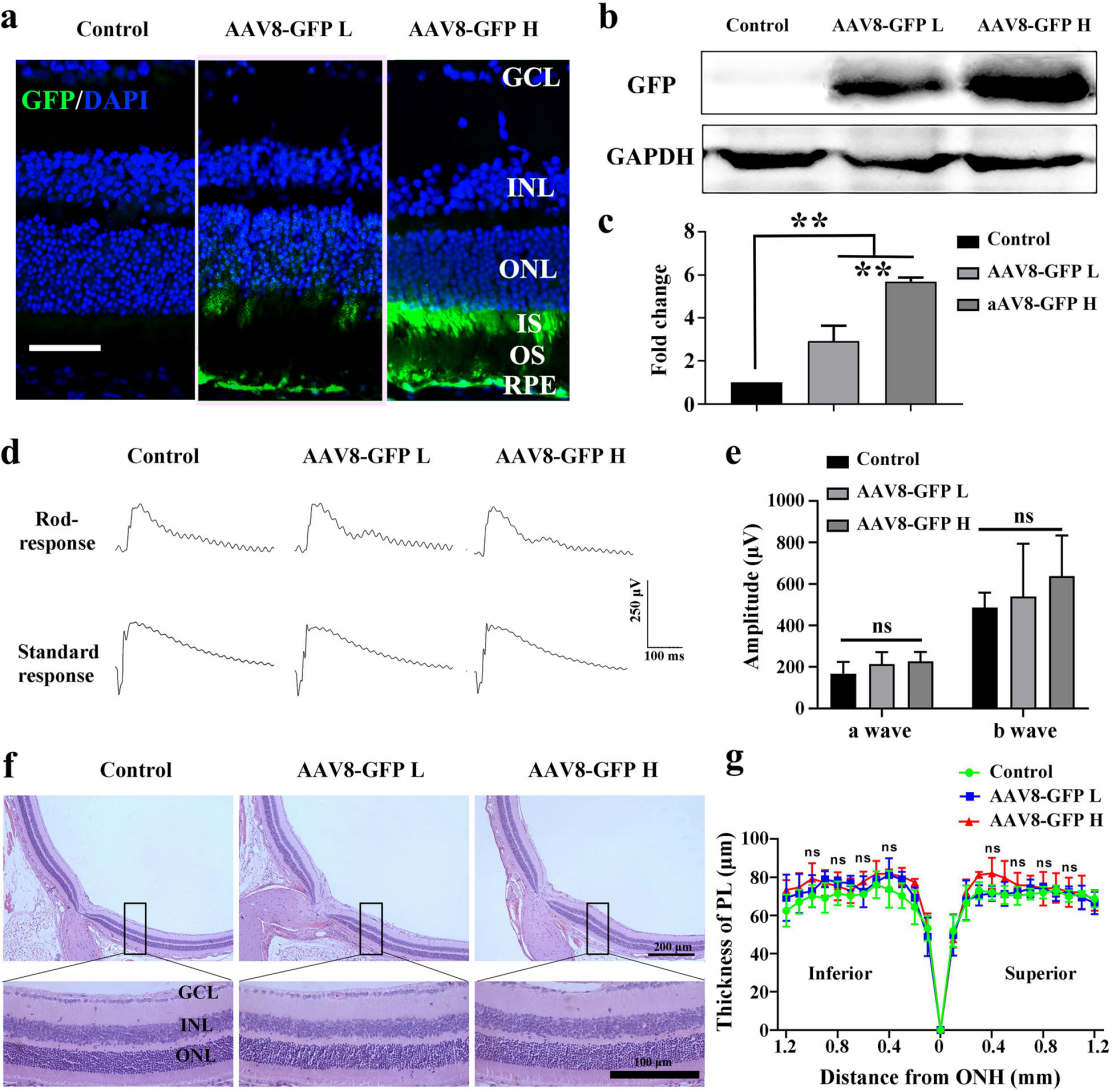
High or low dose of control AAV-GFP virus does not affect retinal structure and function in adult albino mice**. a** GFP immunostaining in retinas from 2-month-old albino mice 2 weeks after infection with a low or a high dose of AAV-GFP. **b** Western blotting of GFP in retinas from albino mice under the indicated conditions. **c** Quantification of GFP expression in retinas according to the above immunoblots (Error bars: SD; *n* = 3, one-way ANOVA). **d** ERG scotopic traces of rod response and standard response from albino mice 2 weeks after infection with low or high dose of AAV8-GFP virus. **e** Quantification of the a- and b-wave amplitudes according to ERG traces of the standard response (Error bars: SD; *n* = 6, one-way ANOVA). **f** Retinal structure of albino mice 2 weeks after infection with a low or high dose of AAV8-GFP virus. **g** Quantification of the thickness of the photoreceptor cell layer according to results of the retinal structure shown in (**f**) (Error bars: SD; *n* = 6, one-way ANOVA). GCL, ganglion cell layer; INL, inner nuclear layer; ONL, outer nuclear layer; PL, photoreceptor layer; ns, no significant difference; * or ** or *** indicates *p* < 0.05 or *p* < 0.01 or *p* < 0.001. Scale bars: 50 μm

There is one caveat, however, with the above mentioned results. The CMV sequence used to regulate gene expression in the recombinant AAV and the dose of the AAV capsid have both been reported to be associated with retinal toxicity [39, 40]. Hence, retinal degeneration induced by the high dose of AAV8-HMOX1 might just be the result of viral toxicity. Therefore, we generated a control AAV8-CMV-GFP virus and injected it at similar doses as the AAV8-HMOX1 (2 × 10^9^ or 5 × 10^8^ viral genomes per eye). Two weeks after infection, both WB and IF data showed a dose-dependent GFP expression in photoreceptor cells (Fig. 4a-c). ERG data showed that the amplitudes of the a and b waves in standard responses were not significantly different between high dose (a wave amplitude: 226.4 ± 46.11; b wave amplitude: 638.5 ± 195.4; *n* = 6) and low dose (a wave amplitude: 213.1 ± 58.23; b wave amplitude: 540.5 ± 253.5; *n* = 6) or control groups (a wave amplitude: 166.8 ± 56.58; b wave amplitude: 486.3 ± 72.76; *n* = 6) (Fig. 4d and e). Moreover, histological data showed that neither high nor low doses of AAV-GFP led to a thinning of the PL compared with control (Fig. 4f and g). These results indicate that overexpression of AAV8-GFP, even at a high dose, does not induce retinal degeneration, suggesting that the retinal degeneration seen after high dose infection with AAV8-HMOX1 is the result of HMOX1 expression and not a toxic side effect of viral infection.

### Excessive HMOX1 leads to mislocalization of phototransduction proteins

**Fig. 5:**
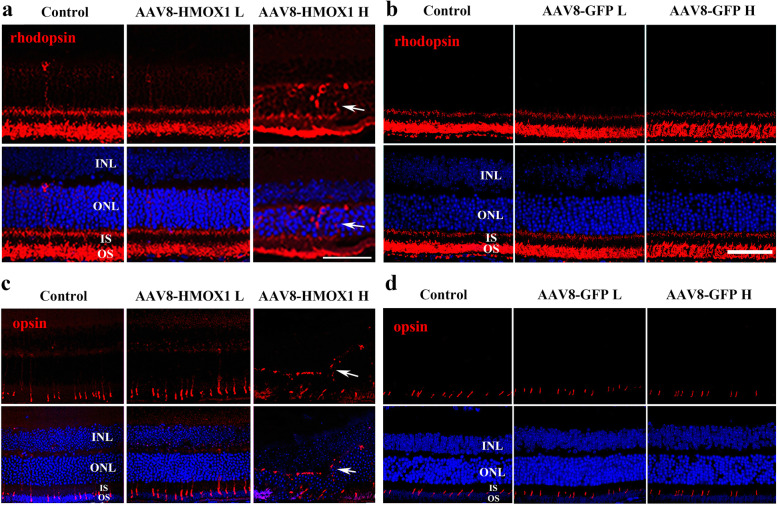
High dose of AAV8-Hmox1 virus causes mislocalization of rhodopsin and opsin. **a, b** Immunostaining of rhodopsin in the retinas from albino mice 2 weeks after infection with a low or high dose of AAV8-GFP (**a**) (*n* = 3) or AAV8-HMOX1 (**b**) (*n* = 3). Note that rhodopsin was abnormally present in the ONL only in the retinas infected with the high dose of AAV8-HMOX1 (arrows). **c, d** Immunostaining of opsin in the retinas from the albino mice 2 weeks after infection with a low or high dose of AAV8-GFP (*n* = 3) or AAV8-HMOX1 (*n* = 3). Note that Opsin was mislocalized in the ONL of the retinas infected with the high dose of AAV8-HMOX1 (arrows). ONL, outer nuclear layer; IS, photoreceptor inner segments; OS, photoreceptor outer segments. Scale bars: 20 μm

To investigate the mechanism by which high dose HMOX1 induces retinal degeneration, we focused on analyzing the trafficking of rhodopsin, because, as shown in Fig. 1, HMOX1induction following light damage is accompanied by rhodopsin mislocalization [41]. As shown in Fig. 5a, rhodopsin protein is normally located at the outer segment layer in neural retinas infected with the low dose of AAV8-HMOX1, but is abnormally present in the ONL of neural retinas infected by the high dose of AAV8-HMOX1. This mislocalization of rhodopsin to the ONL was not observed in neural retinas infected with either the high or the low dose of AAV8-GFP (Fig. 5b). These results suggest that the excessive HMOX1 disrupts trafficking of rhodopsin to the outer segment layer of rod cells.

To further investigate whether the high dose of AAV8-HMOX1 affects protein trafficking, we examined the localization of cone opsin. Similarly, as shown in Fig. 5c, opsin protein was also mislocalized at the ONL of retinas infected by the high dose of AAV8-HMOX1. As expected, in neural retinas infected by the two doses of AAV8-GFP, opsin was normally localized at the inner segment layer (Fig. 5d). These data indicate that excessive HMOX1 disturbs proper localization of phototransduction proteins at the OS of rod cells and inner segment of cone cells.

### HMOX1 regulates the expression of the ER stress effector *Ddit3* in retinas

**Fig. 6:**
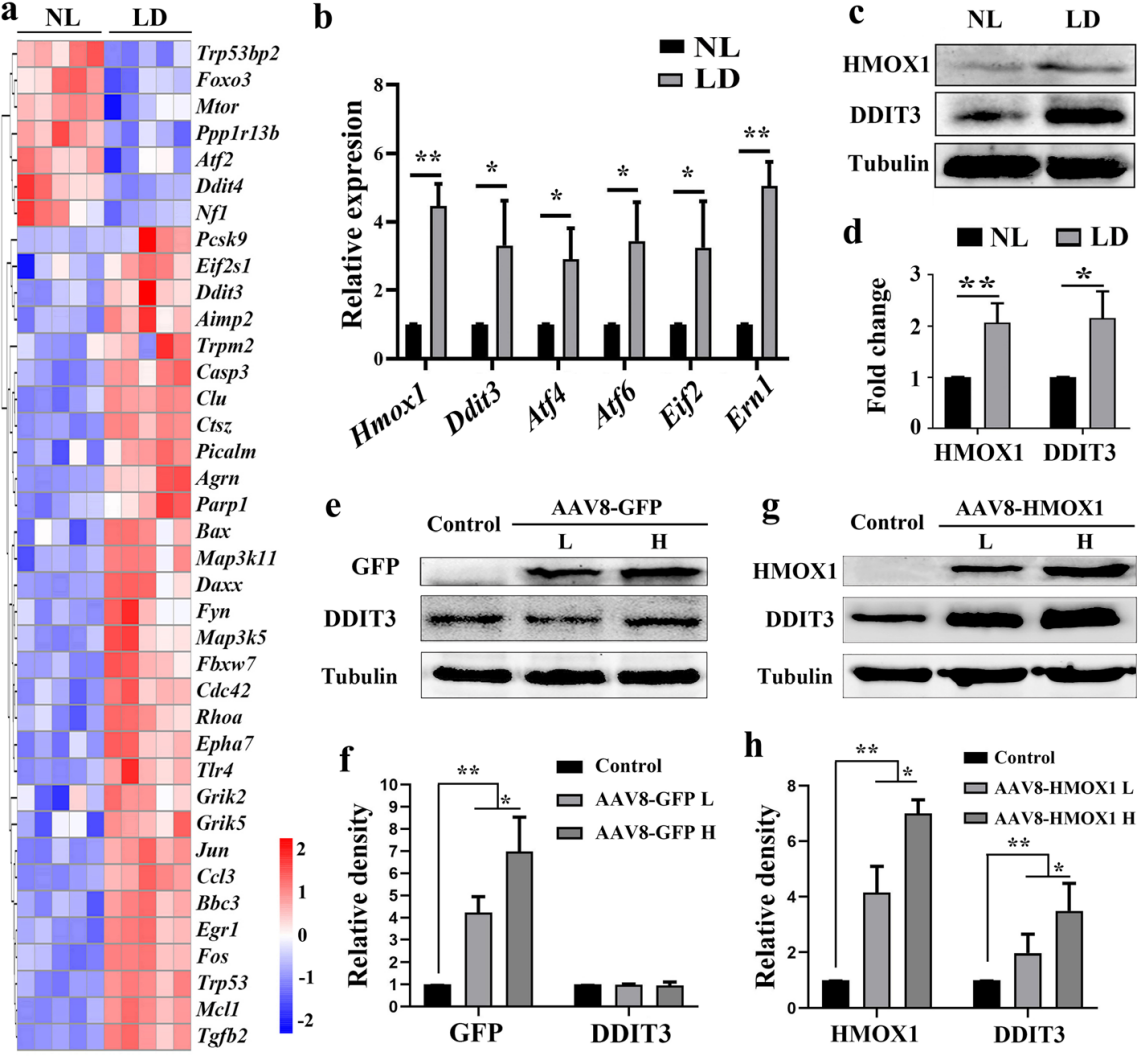
HMOX1 positively regulates *Ddit3* expression**. a** Heat-map of differentially expressed genes from the cluster of positive neuron cell death regulator genes based on the GO analysis of the RNA seq data. Results from albino neural retinas after LD for 1 day (*n* = 5, *p* < 0.05, one-way ANOVA) (NL: normal light control). **b** q-RT-PCR data showing expression of Hmox1 and ER stress-related genes in albino neural retinas after 1 day of LD (Error bars: SD; *n* = 3, Student *T*-test). **c, d** Western blots and corresponding quantification using anti-HMOX1 and anti-DDIT3 in neural retinas after LD for 1 day (Error bars: SD; *n* = 3, one-way ANOVA). **e-h** Western blots of anti-GFP and anti-DDIT3 in neural retinas infected by a low (*n* = 3) or high dose (*n* = 3) of AAV8-GFP (**e**) and quantitative bar graphs according to the immunoblots (**f**) (Error bars: SD; one-way ANOVA). **g, h** Western blots of anti-HMOX1 and anti-DDIT3 in retinas infected by a low (*n* = 3) or high (*n* = 3) dose of AAV-HMOX1 **(g**) and quantitative bar graphs of expression of HMOX1 and DDIT3 (**h**) (Error bars: SD; one-way ANOVA). * or ** indicates *p* < 0.05 or *p* < 0.01

To elucidate the molecular mechanism of retinal degeneration caused by excessive HMOX1 levels, we re-analyzed our previous RNA-seq transcriptome data (GSE146176) from neural retinas of albino mice kept under normal or high intensity light conditions [30]. As shown in Fig. 6a, GO analysis allowed assignment of about 30 differentially expressed genes to the category of pro-apoptotic genes. Among these genes, *Ddit3,* an ER stress effector known to trigger cell death, was upregulated after light exposure as previously reported [42]. Quantitative-RT-PCR data confirmed that after 1 day of LD, both *Hmox1* and some ER stress-related genes including *Ddit3* were upregulated (Fig. 6b). WB data also showed that after 1 day of LD, DDIT3 expression was upregulated (2.15 ± 0.52 fold, *n* = 3) along with the induction of HMOX1 (2.07 ± 0.37 fold, *n* = 3) (Fig. 6c and d). These results indicate that the *Ddit3* expression is correlated with the induction of *Hmox1* during LD.

To analyze whether overexpression of HMOX1 might induce *Ddit3* expression in the unstressed condition, we examined *Ddit3* expression in neural retinas 2 weeks after infection with AAV8-HMOX1. Q-RT-PCR data showed that indeed, AAV8-HMOX1, but not AAV8-GFP, upregulated *Ddit3* expression as well as other ER stress-related genes in a dose-dependent manner (Additional file 3: Figure S3). These results were supported by WB analysis (Fig. 6e-h), suggesting that HMOX1 positively regulates *Ddit3* expression in neural retinas.

A tight regulation between iron deposits and ER stress has been reported [43]. Therefore, we aimed to study whether HMOX1-induced ER stress might be related to its catalyzed metabolite iron. As shown in Additional file 4, a significant increase in non-heme iron was detected in neural retinas infected by AAV8-HMOX1 (Additional file 4: Figure S4a and c) instead of AAV8-GFP (Additional file 4: Figure S4b and d), and the level of iron was increased with the dose of AAV8-HMOX1. We then used FAC as the iron source to analyze whether iron-overload induces DDIT3 expression in photoreceptor cells. The data showed that > 50 μM of FAC can induce expression of DDIT3 and its upstream regulator ATF4, and the increases of DDIT3 by FAC were dose dependent (Additional file 5: Figure S5a and b). Collectively, these data suggest that AAV8-HMOX1 can upregulate DDIT3 through iron-overload in photoreceptor cells.

### Genetic deletion of *Ddit3* prevents retinal degeneration caused by the excessive HMOX1

**Fig. 7:**
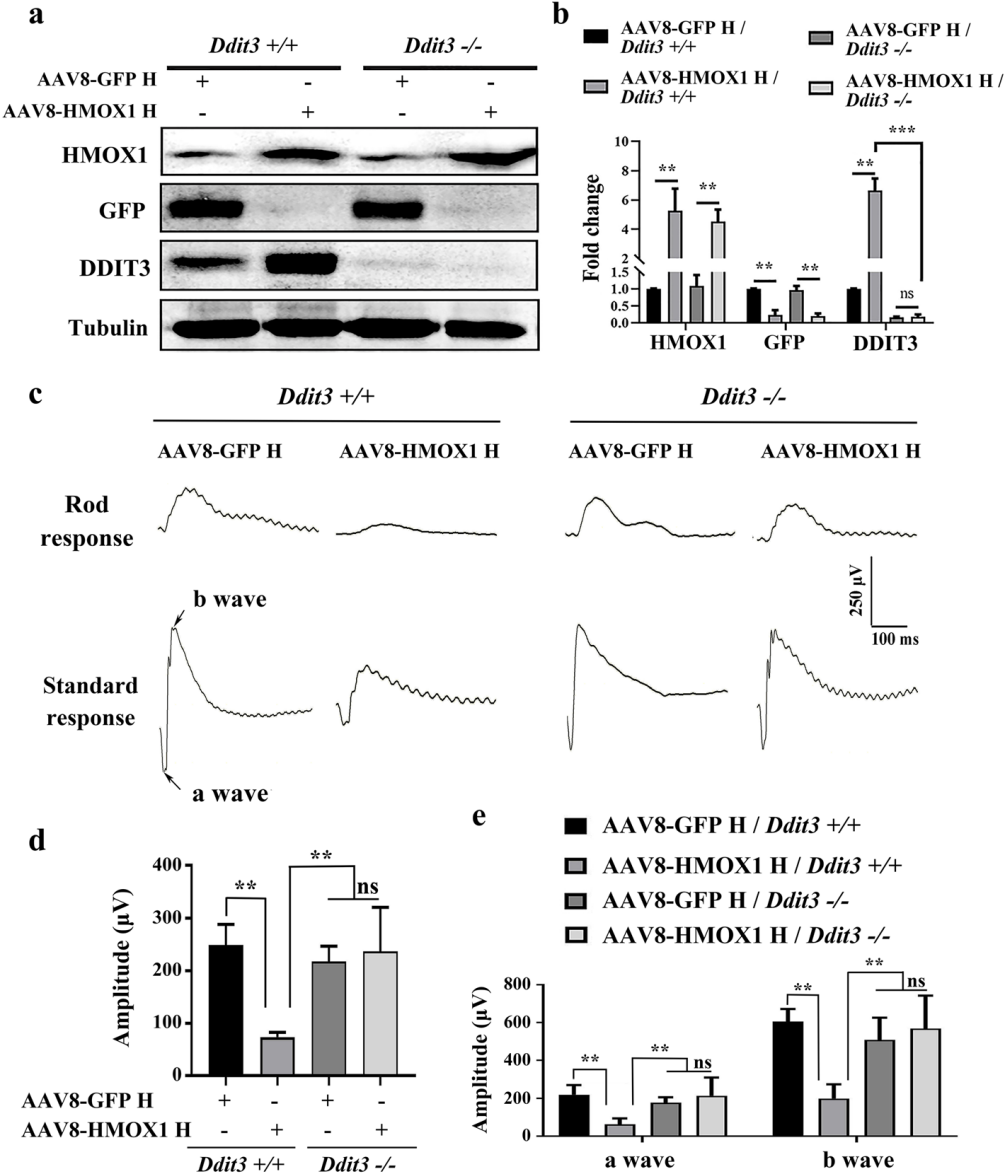
Deletion of *Ddit3* prevents abnormal retinal function induced by the AAV8-mediated high dose of HMOX1**. a, b** Western blots of neural retinas from *Ddit3+/+* (*n* = 3) and *Ddit3−/−*mice (*n* = 3) 2 weeks after infection with a high dose of AAV8-GPF or AAV8-HMOX1, using anti-HMOX1, anti-GFP or anti-DDIT3 antibodies (**a**) and corresponding quantitative bar graphs (**b**) (Error bars: SD; one-way ANOVA; *, *p* < 0.05; **, *p* < 0.01; ***p < 0.001). **c** ERG traces of 2-month-old *Ddit3+/+* (left panels) and *Ddit3−/−* (right panels) mice 2 weeks after infection with the high dose of AAV8-GFP or AAV8-HMOX1. **d, e** Quantification of ERG amplitudes in rod response (**d**) and standard response (**e**) according to the ERG trace results from c (Error bars: SD; *n* = 4, one-way ANOVA; *, *p* < 0.05; **, *p* < 0.01)

Based on the fact that retinal degeneration associated with high dose of HMOX1 levels is correlated with upregulation of *Ddit3* expression, we finally evaluated whether the induction of DDIT3 might mediate HMOX1-induced photoreceptor degeneration. To address this question, 2-month-old *Ddit3−/−* mice were infected by the high dose of AAV8-HMOX1 for 2 weeks and then subjected to retinal function and degeneration analysis. WB data showed that after 2 weeks of infection, AAV8-GFP or AAV8-HMOX1 correspondingly induced high expression of GFP (4.73 ± 1.01fold, *n* = 3) or HMOX1 (5.26 ± 1.52 fold, *n* = 3) in both *Ddit3+/+* and *Ddit3−/−* neural retinas (Fig. 7a and b). As expected, AAV8-HMOX1 induced DDIT3 expression in the *Ddit3+/+* neural retinas (6.65 ± 0.82 fold, *n* = 3), but not in the *Ddit3−/−* neural retinas (Fig. 7a and b).

**Fig. 8:**
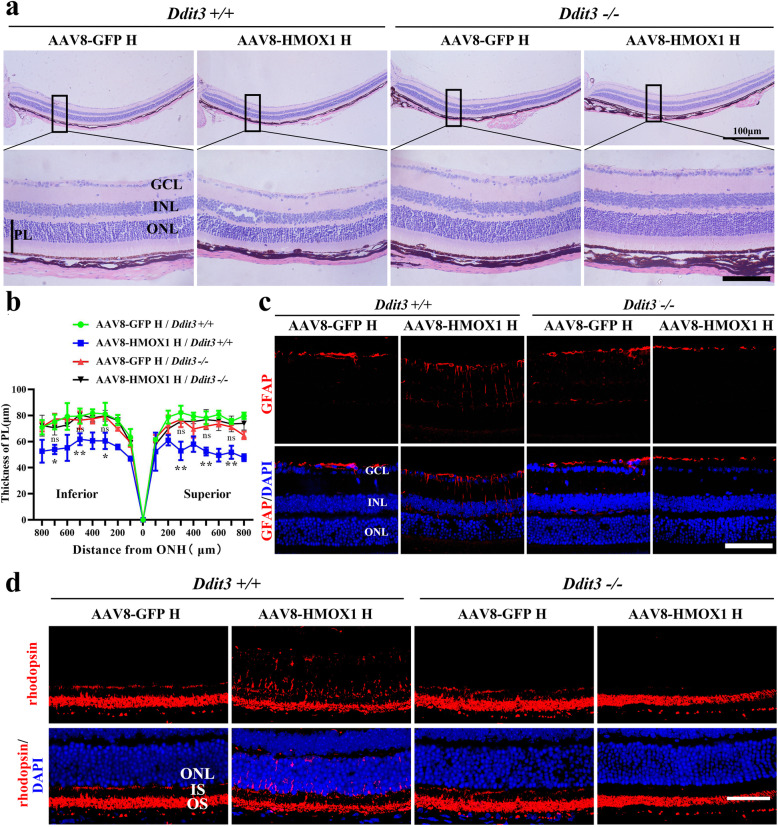
Deletion of *Ddit3* prevents photoreceptor degeneration caused by the excessive HMOX1 levels**. a, b** Histological images of H&E staining from *Ddit3+/+*(*n* = 3) and *Ddit3−/−* mice (*n* = 3) 2 weeks after infection with a high dose of AAV8-GPF or AAV8-HMOX1 (**a**) and curve diagram of PL thickness according to the H&E staining (**b**) (Error bars: SD; one-way ANOVA; *, *p* < 0.05; **, *p* < 0.01; ***, *p* < 0.001;). **c, d** Immunostaining images of anti-GFAP or anti rhodopsin in retinas from *Ddit3+/+* (*n* = 3) and *Ddit3−/−* (*n* = 3) mice under the indicated conditions. GCL, ganglion cell layer; INL, inner nuclear layer; ONL, outer nuclear layer; PL, photoreceptor layer; ns, no significant difference. * or ** or *** indicates *p* < 0.05 or *p* < 0.01 or *p* < 0.001. Scale bars: 50 μm

We then analyzed the corresponding retinal functions by ERG examination. We found that the standard responses under unstressed conditions were similar in *Ddit3+/+* and *Ddit3−/−* mice (a wave amplitude: 144.8 ± 27.02 versus 172.5 ± 38.21, respectively; b wave amplitude: 435.1 ± 74.8 versus 498.3 ± 112.6, respectively; *n* = 4 for each genotype) (Additional file 6: Figure S6). Injection of a high dose of AAV8-HMOX1, however, led to a significant decline of the amplitudes in *Ddit3+/+* mice (a wave amplitude: 62.5 ± 31.32; b wave amplitude: 199.1 ± 75.29; *n* = 4), but not in *Ddit3−/−* mice (a wave amplitude: 213.9 ± 94.69; b wave amplitude: 567.9 ± 175.4; *n* = 4) in standard responses. Injection of a high dose of control AAV8-GFP affected the a and b waves in neither *Ddit3+/+* nor *Ddit3−/−*mice (Fig. 7c-e). These results suggest that *Ddit3* mediates the reduced retinal function following high level expression of HMOX1. This interpretation is supported by the fact that among the four experimental groups, photoreceptor degeneration and cell death were only observed in the *Ddit3+/+* neural retinas infected by the high dose of AAV8-HMOX1 (Fig. 8a and b and Additional file 7: Figure S7). In addition, the injection of AAV8-HMOX1 only induced upregulation of GFAP (Fig. 8c) and mislocalization of rhodopsin (Fig. 8d) in neural retinas of *Ddit3+/+* mice but not in those of *Ddit3−/−*mice. Taken together, these results indicate that deletion of *Ddit3* blocks the effect of AAV8-HMOX1-induced photoreceptor degeneration, suggesting that DDIT3 is involved in excessive HMOX1-triggered photoreceptor degeneration.

## Discussion

It is well established that HMOX1 can play beneficial as well as detrimental roles in neurodegenerative diseases [9, 16, 17, 30, 31]. Using a mouse model of light-induced retinal degradation, we here show that the distinction of whether HMOX1 is beneficial or detrimental depends on the level of HMOX1 induction. Thus, a low level of HMOX1 expression as achieved by AAV-mediated gene transfer at a low dose protects against light-induced retinal degeneration while a high level of HMOX1 expression after high dose gene transfer leads to retinal degeneration. We further show that photoreceptor degeneration induced by a high dose of HMOX is mediated by regulation of the ER stress-inducing factor DDIT3. Hence, these findings support the notion that in addition to its known antioxidant function, HMOX1 may trigger ER stress and so lead to photoreceptor death rather than to protection from oxidative damage.

We find the results summarized above are compelling mainly based on using appropriate AAV-GFP controls at corresponding high and low doses and a variety of internally consistent assays. Nevertheless, the study leaves us with two big questions. First, what exactly is the threshold between the two doses, and at that threshold level, would beneficial and detrimental effects cancel each other out? A determination of the threshold may be difficult to achieve in vivo, however, given that there may be region-specific variations in HMOX1 expression levels and that the time periods over which the effects have to be observed might not allow to continuously maintain expression levels. The second question concerns the mechanism of induction of DDIT3. The results have clearly indicated that the ER stress factor DDIT3 is correlated with HMOX1 induction, that HMOX1 induces DDIT3 in the absence of experimental light damage, and that DDIT3 is required for the manifestations of the detrimental effects of HMOX1. But how does HMOX1 induce DDIT3?

**Fig. 9:**
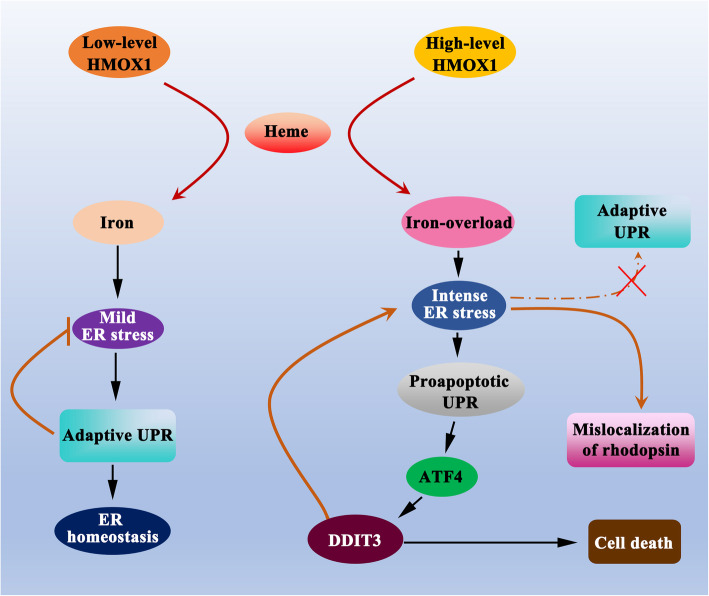
A potential mechanism of the dual roles of HMOX1 in neural retinas. Low-levels of HMOX1 reduce oxidative stress by catalyzing the degradation of heme to biliverdin, CO and ferrous iron because of the consumption of molecular oxygen in the heme oxygenase reaction. Although ferrous iron leads to mild ER stress, the adaptive UPR likely restores ER homeostasis. In contrast, high-levels of HMOX1 induce intense ER stress via iron-overload, resulting in protein unfolding or misfolding in the dysfunctional ER, which in turn leads to mislocalization of rhodopsin in photoreceptor cells. The intense ER stress, rather than inducing an adaptive UPR, triggers a proapoptotic UPR that leads to cell death via the stimulation of the DDIT3 pathway. Thus, the level of ferrous iron might represent the switching point determining whether HMOX1 plays a protective or a deleterious role in neural retinas

Answers to this second question may come from considering the physiological roles of HMOX1 in cells. HMOX1 catalyzes heme to biliverdin, CO and ferrous iron and is normally anchored in the ER, although it is also found in other subcellular localizations [7]. The catalyzed metabolite ferrous iron is an essential element for neuronal functions, while iron overload has been known to cause neuronal toxicity through ER stress [43]. Besides, deletion of *Hmox1* in microglia blocks iron overload-induced neuronal toxicity [34]. Excess HMOX1 may hence lead to ER stress via iron overload, which in turn may trigger the unfolded protein response (UPR) with the concomitant upregulation of PERK (protein kinase RNA-like ER kinase), ATF4 and ATF6 (activating transcription factor 4 and 6) and IRE1 (inositol-requiring enzyme 1) [22, 44]. With increasing levels of ER stress, however, the originally anti-apoptotic function of the adaptive UPR may switch to a pro-apoptotic function by inducing caspases and DDIT3, the latter along with other ER-stress-related genes normally expressed at low levels but upregulated in response to severe or persistent ER perturbations [44, 45] as schematically illustrated in Fig. 9. Future investigations may provide experimental evidence for this scenario, but it is important at this point to note that the concept is different from the well known role of HMOX1 as an anti-oxidant factor.

The above mentioned two questions and speculations then lead to a third question, namely is the protective role of HMOX1 following low dose expression, which is likely linked to its anti-oxidative function, simply the consequence of a lack of induction of high level ER-stress and the corresponding set of ER-stress-related genes? Such an interpretation is certainly supported by the fact that the low dose of HMOX1 expression as presented in this study does not affect the ER residency of HMOX1 in the inner segment of photoreceptors and preserves photoreceptor cell survival after light damage. It is further supported by the above finding that in the absence of DDIT3, even high levels of HMOX1 expression do not lead to photoreceptor degeneration. In addition to our use of AAV-GFP, this finding likewise argues against a potential retinal toxicity of the viral vector [39, 40], which has been described for doses of 5 billion viral genomes per eye [40] as opposed to our high dose of 2 billion viral genomes per eye. Hence, photoreceptor degeneration caused by our high dose of HMOX1 can safely be attributed to the excessive HMOX1 expression, which in turn triggers an ER stress response above a tolerance level. It remains to be shown in detail, however, how HMOX1 regulates DDIT3 in the course of light-induced and potentially other forms of retinal damage.

Among the many mechanisms that may be responsible for HMOX1-mediated retinal neurodegeneration, we were intrigued by the finding that excessive HMOX1 expression was associated with rhodopsin mislocalization. Rhodopsin is a prototypical G protein-coupled receptor (GPCR) responsible for light sensing and is synthesized and folded in the endoplasmic reticulum [46]. Mutations in *RHO* are common causes of heritable Retinitis pigmentosa (RP) [47], and some of its coding mutations, such as P23H, T17M and V20G, lead to rhodopsin mistrafficking [41]. The underlying mechanisms are thought to be associated with the inability of mutant rhodopsins to properly fold in the ER, which might lead to ER stress [41, 48]. Nevertheless, as also observed in our study, rhodopsin mislocalization may occur during photoreceptor degeneration independent of mutations. Conceivably, the ER stress effector *Ddit3* may block wildtype rhodopsin from being normally translocated, while it is not required for mislocalization of mutant rhodopsin [49]. Therefore, although rhodopsin mutations can definitely trigger rhodopsin mislocalizations and ER stress, the reverse, that ER stress can lead to rhodopsin mislocalization, is equally possible.

## Conclusions

Our data confirm previous studies that HMOX1 protects photoreceptor cells from light-induced retinal degeneration. We here extend these findings by showing that excessive expression of HMOX1 may also trigger ER stress and not simply be its consequence, and that DDIT3 is the major mediator of the pro-apoptotic role of excessive HMOX1. Therefore, HMOX1 expression levels should be considered when treating retinal neurodegenerative diseases because it is these levels that determine whether or not DDIT3 accumulates at detrimental heights. Hence, these findings have implications for pharmacological approaches that target DDIT3 as a potential way to eliminate the detrimental side effects of HMOX1 induction and so promote cell survival in retinal neurodegenerative diseases.

## Supplementary Information

**Additional file 1 **: **Figure S1.** Photoreceptor degeneration is aggravated with increasing duration of light exposure**. (A)** Representative histological H&E staining of retinas from the 2-month-old albino mice exposed to LD. **(B)** The curve diagram shows the thickness of PL of mice kept under the indicated conditions (Error bars: SD; *n* = 6, one-way ANOVA). (**C**) Representative immunostaining images for GFAP in retinas from albino mice exposed to high-intensity light for the indicated times. (**D**) Representative images of TUNEL assays of retinas of albino mice exposed to high-intensity light for the indicated times. (**E**) Quantification of cell death in the ONL of the retinas from albino mice kept under the indicated conditions (Error bars: SD; *n* = 3, one-way ANOVA). GCL, ganglion cell layer; INL, inner nuclear layer; ONL, outer nuclear layer; PL, photoreceptor layer. * or ** or *** indicates *p* < 0.05 or *p* < 0.01 or *p* < 0.001. Scale bar: 50 μm.

**Additional file 2 **: **Figure S2.** AAV8-mediated low level expression of HMOX1 protects photoreceptor cells from LD. 2-month-old albino mice were infected by the low dose of AAV8-HMOX1 or control virus for 2 weeks, then exposed to high-intensity light (15,000 lx) for 3 days and finally analyzed for retinal degeneration. **(A)** ERG traces of albino mice infected with the low dose of AAV8-HMOX1 or control virus after 3 days of LD. (**B, C**) Quantification of ERG amplitudes in rod response (**B**) and standard response (**C**) according to the ERG traces (Error bars: SD; *n* = 6, Student *T*-test). (**D**) Representative histological images of H&E staining of retinas infected with the indicated viruses, kept for 2 weeks and then exposed to high intensity light continuously for 3 days. (**E**) The curve diagram of the thickness of photoreceptor cell layer from the albino mice kept under the indicated conditions (Error bars: SD; *n* = 6, one-way ANOVA). (**F, G**) Representative images of TUNEL assays from retinas of albino mice exposed to high-intensity light for the indicated times (**F**) and the quantification of cell death in the ONL of retinas (**G**) (Error bars: SD; *n* = 3, Student *T*-test). GCL, ganglion cell layer; INL, inner nuclear layer; ONL, outer nuclear layer. PL, photoreceptor layer. * or ** indicates *p* < 0.05 or *p* < 0.01. Scale bar: 50 μm.

**Additional file 3 **: **Figure S3.** AAV8-HMOX1 up-regulates expressions of ER stress-related genes. 2-month-old albino mice were infected by the indicated virus and after 2 weeks subjected to gene expression analysis. **(A)** Quantification of q-RT-PCR shows expression of *Gfp* and other ER stress-related genes in the retinas infected with the low or the high dose of AAV8-GFP (Error bars: SD; *n* = 3, one-way ANOVA). **(B)** Quantification of q-RT-PCR shows expression of *Hmox1* and ER stress-related genes in retinas infected by the low or the high dose of AAV8-HMOX1 after 2 weeks (Error bars: SD; *n* = 3, one-way ANOVA). Note that upregulation of ER stress-related genes by AAV8-HMOX1 is dose-dependent. * or ** or *** indicates *p* < 0.05 or *p* < 0.01 or *p* < 0.001.

**Additional file 4 **: **Figure S4.** AAV8-HMOX1 increases the level of non-heme iron in neural retinas. 2-month-old albino mice were infected with the indicated virus and after 2 weeks subjected to non-heme iron examination. (**A, B**) Images of chromogenic reaction solution from retinas infected with a low or high dose of AAV8-HMOX1 (A) or AAV8-GFP (B). “Reagent” corresponds to the reaction solution, and “Control” to the result obtained with neural retinas without virus infection. (**C, D**) Quantification of the relative level of non-heme iron in the retinas infected with the low or the high dose of AAV8-HMOX1 (C) or AAV8-GFP (D) (Error bars: SD; *n* = 5, one-way ANOVA). Note that increase of non-heme iron by AAV8-HMOX1 is dose-dependent. ** indicates *p* < 0.01.

**Additional file 5 **: **Figure S5.** Iron-overload induces expressions of DDIT3 and ATF4 in photoreceptor cells**. (A)** 661 W photoreceptor cells were stimulated by the indicated doses of FAC (ferric ammonium citrate) and DDIT3 and ATF4 were determined using corresponding antibodies. (**B)** Quantification based on Western blot results shown in A (Error bars: SD; one-way ANOVA; *n* = 3; **, *p* < 0.01). Note that ER stress induced by FAC is dose-dependent in photoreceptor cells.

**Additional file 6 **: **Figure S6.** Deletion of *Ddit3* does not disrupt retinal function**. (A)** ERG traces of 2-month-old *Ddit3+/+* (left panels) and *Ddit3−/−* mice (right panels) in rod response and standard response**. (B, C)** Quantification of ERG amplitudes in rod response (**B**) and standard response (**C**) according to the results form B (Error bars: SD; *n* = 4, Student *T*-test).

**Additional file 7 **: **Figure S7.** Deletion of *Ddit3* prevents photoreceptor cell death induced by the AAV8-mediated high dose of HMOX1. (**A**) Representative images of TUNEL assays from 2-month-old *Ddit3+/+* (left panels) and *Ddit3−/−* (right panels) retinas 2 weeks after infection with the high dose of AAV8-GFP (upper panels) or of AAV8-HMOX1 (lower panels). (**B**) Quantification of cell death in the ONL of the *Ddit3+/+* and *Ddit3−/−* retinas infected with the high dose of AAV8-GFP or of AAV8-HMOX1 (Error bars: SD; *n* = 4, one-way ANOVA). Note that deletion of *Ddit3* rescues photoreceptor cell death caused by the high dose of AAV8-HMOX1. INL, inner nuclear layer; ONL, outer nuclear layer. ** indicates *p* < 0.01. Scale bar: 50 μm.

**Additional file 8 **: **Table 1**. Sequences of primers used for gene expression.

## Data Availability

The datasets and materials used and/or analyzed during the current study are available from the corresponding author on reasonable request.

## Abbreviations

AAV: Adeno-associated virus
AD: Alzheimer’s disease
AMD: Age-related macular degeneration
ATF4: Activating transcription factor 4
ATF6: Activating transcription factor 6
CHOP: C/EBP-homologous protein
CMV: Cytomegalovirus
CNS: Central nerves system
CO: Carbon monoxide
DDIT3: DNA damage inducible transcript 3
ER: Endoplasmic reticulum
ERG: Electroretinography
GCL: Ganglion cell layer
GFP: Green Fluorescent Protein
GO: Gene ontology
GPCR: G protein-coupled receptor
H&E staining: Hematoxylin-Eosin staining
Hmox1: Heme oxygenase-1
IF: Immunofluorescence
INL: Inner nuclear layer
IRE1: Inositol-requiring enzyme 1
IS: Inner segment layer
LD: Light damage
LIRD: Light-induced retinal degeneration
ONL: Outer nuclear layer
OS: Outer segment layer
PERK: Protein kinase RNA-like ER kinase
PD: Parkinson’s disease
PL: Photoreceptor cell layer
RP: Retinitis pigmentosa
TUNEL assay: Terminal deoxynucleotidyl transferase-mediated dUTP-biotin nick end labeling assay
UPR: Unfolded protein response
WB: Western blot
