# Supplementary material for: High dose expression of heme oxigenase-1 induces retinal degeneration through ER stress-related DDIT3
Source: Mol Neurodegener. 2021 Mar 10;16:16. doi: 10.1186/s13024-021-00437-4 (PMC7944639; doi:10.1186/s13024-021-00437-4)
Supplement: Supplementary file 1 — Additional file 1 : Figure S1. Photoreceptor degeneration is aggravated with increasing duration of light exposure. (A) Representative histological H&E staining of retinas from the 2-month-old albino mice exposed to LD. (B) The curve diagram shows the thickness of PL of mice kept under the indicated conditions (Error bars: SD; n = 6, one-way ANOVA). (C) Representative immunostaining images for GFAP in retinas from albino mice exposed to high-intensity light for the indicated times. (D) Representative images of TUNEL assays of retinas of albino mice exposed to high-intensity light for the indicated times. (E) Quantification of cell death in the ONL of the retinas from albino mice kept under the indicated conditions (Error bars: SD; n = 3, one-way ANOVA). GCL, ganglion cell layer; INL, inner nuclear layer; ONL, outer nuclear layer; PL, photoreceptor layer. * or ** or *** indicates p < 0.05 or p < 0.01 or p < 0.001. Scale bar: 50 μm. [file 13024_2021_437_MOESM1_ESM.docx]

**
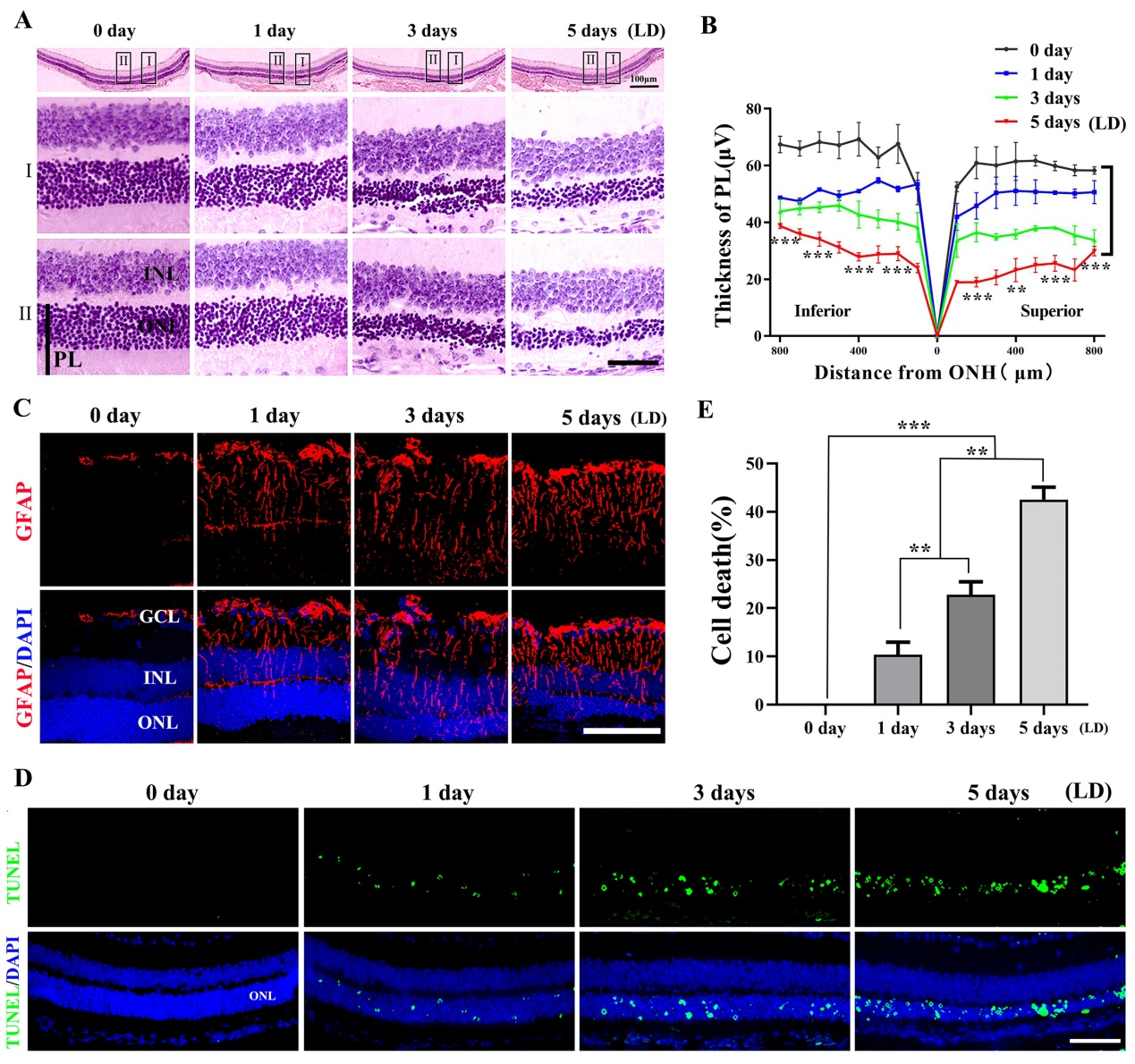
**

**Additional file 1:**

**Figure S1.** Photoreceptor degeneration is aggravated with increasing duration of light exposure**. (A)** Representative histological H&E staining of retinas from the 2-month-old albino mice exposed to LD. **(B)** The curve diagram shows the thickness of PL of mice kept under the indicated conditions (Error bars: SD; n=6, one-way ANOVA). (**C**) Representative immunostaining images for GFAP in retinas from albino mice exposed to high-intensity light for the indicated times. (**D**) Representative images of TUNEL assays of retinas of albino mice exposed to high-intensity light for the indicated times. (**E**) Quantification of cell death in the ONL of the retinas from albino mice kept under the indicated conditions (Error bars: SD; n=3, one-way ANOVA). GCL, ganglion cell layer; INL, inner nuclear layer; ONL, outer nuclear layer; PL, photoreceptor layer. * or ** or *** indicates p<0.05 or p<0.01 or p<0.001. Scale bar: 50 μm.
