# Supplementary material for: High dose expression of heme oxigenase-1 induces retinal degeneration through ER stress-related DDIT3
Source: Mol Neurodegener. 2021 Mar 10;16:16. doi: 10.1186/s13024-021-00437-4 (PMC7944639; doi:10.1186/s13024-021-00437-4)
Supplement: Supplementary file 2 — Additional file 2 : Figure S2. AAV8-mediated low level expression of HMOX1 protects photoreceptor cells from LD. 2-month-old albino mice were infected by the low dose of AAV8-HMOX1 or control virus for 2 weeks, then exposed to high-intensity light (15,000 lx) for 3 days and finally analyzed for retinal degeneration. (A) ERG traces of albino mice infected with the low dose of AAV8-HMOX1 or control virus after 3 days of LD. (B, C) Quantification of ERG amplitudes in rod response (B) and standard response (C) according to the ERG traces (Error bars: SD; n = 6, Student T-test). (D) Representative histological images of H&E staining of retinas infected with the indicated viruses, kept for 2 weeks and then exposed to high intensity light continuously for 3 days. (E) The curve diagram of the thickness of photoreceptor cell layer from the albino mice kept under the indicated conditions (Error bars: SD; n = 6, one-way ANOVA). (F, G) Representative images of TUNEL assays from retinas of albino mice exposed to high-intensity light for the indicated times (F) and the quantification of cell death in the ONL of retinas (G) (Error bars: SD; n = 3, Student T-test). GCL, ganglion cell layer; INL, inner nuclear layer; ONL, outer nuclear layer. PL, photoreceptor layer. * or ** indicates p < 0.05 or p < 0.01. Scale bar: 50 μm. [file 13024_2021_437_MOESM2_ESM.docx]

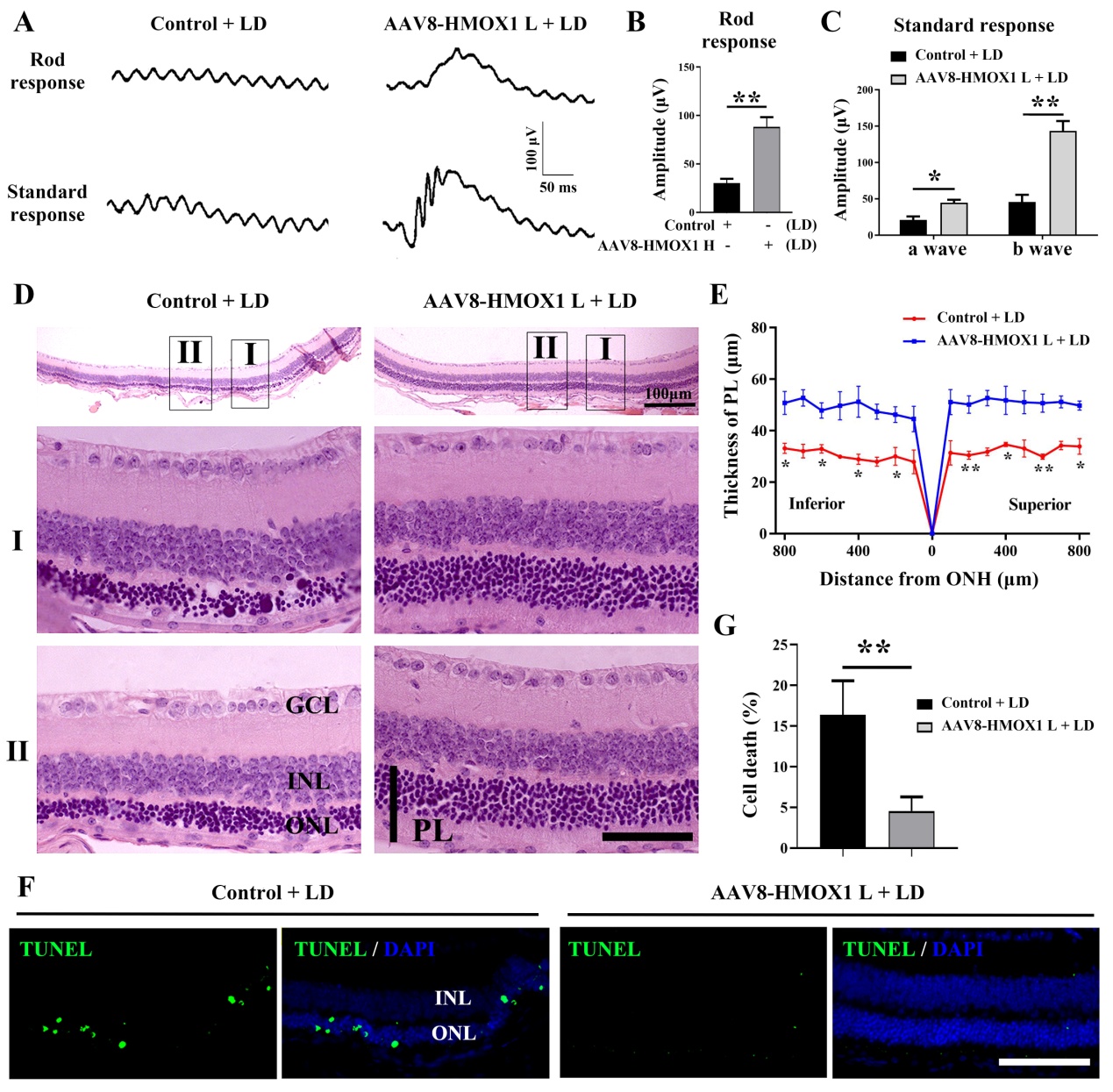


**Additional file 2:**

**Figure S2.** AAV8-mediated low level expression of HMOX1 protects photoreceptor cells from LD**.** 2-month-old albino mice were infected by the low dose of AAV8-HMOX1 or control virus for 2 weeks, then exposed to high-intensity light (15,000 lux) for 3 days and finally analyzed for retinal degeneration. **(A)** ERG traces of albino mice infected with the low dose of AAV8-HMOX1 or control virus after 3 days of LD. (**B, C**) Quantification of ERG amplitudes in rod response (**B**) and standard response (**C**) according to the ERG traces (Error bars: SD; n=6, Student *T*-test). (**D**) Representative histological images of H&E staining of retinas infected with the indicated viruses, kept for 2 weeks and then exposed to high intensity light continuously for 3 days. (**E**) The curve diagram of the thickness of photoreceptor cell layer from the albino mice kept under the indicated conditions (Error bars: SD; n=6, one-way ANOVA). (**F, G**) Representative images of TUNEL assays from retinas of albino mice exposed to high-intensity light for the indicated times (**F**) and the quantification of cell death in the ONL of retinas (**G**) (Error bars: SD; n=3, Student *T*-test). GCL, ganglion cell layer; INL, inner nuclear layer; ONL, outer nuclear layer. PL, photoreceptor layer. * or ** indicates p<0.05 or p<0.01. Scale bar: 50 μm.
