# Supplementary material for: High dose expression of heme oxigenase-1 induces retinal degeneration through ER stress-related DDIT3
Source: Mol Neurodegener. 2021 Mar 10;16:16. doi: 10.1186/s13024-021-00437-4 (PMC7944639; doi:10.1186/s13024-021-00437-4)
Supplement: Supplementary file 3 — Additional file 3 : Figure S3. AAV8-HMOX1 up-regulates expressions of ER stress-related genes. 2-month-old albino mice were infected by the indicated virus and after 2 weeks subjected to gene expression analysis. (A) Quantification of q-RT-PCR shows expression of Gfp and other ER stress-related genes in the retinas infected with the low or the high dose of AAV8-GFP (Error bars: SD; n = 3, one-way ANOVA). (B) Quantification of q-RT-PCR shows expression of Hmox1 and ER stress-related genes in retinas infected by the low or the high dose of AAV8-HMOX1 after 2 weeks (Error bars: SD; n = 3, one-way ANOVA). Note that upregulation of ER stress-related genes by AAV8-HMOX1 is dose-dependent. * or ** or *** indicates p < 0.05 or p < 0.01 or p < 0.001. [file 13024_2021_437_MOESM3_ESM.docx]

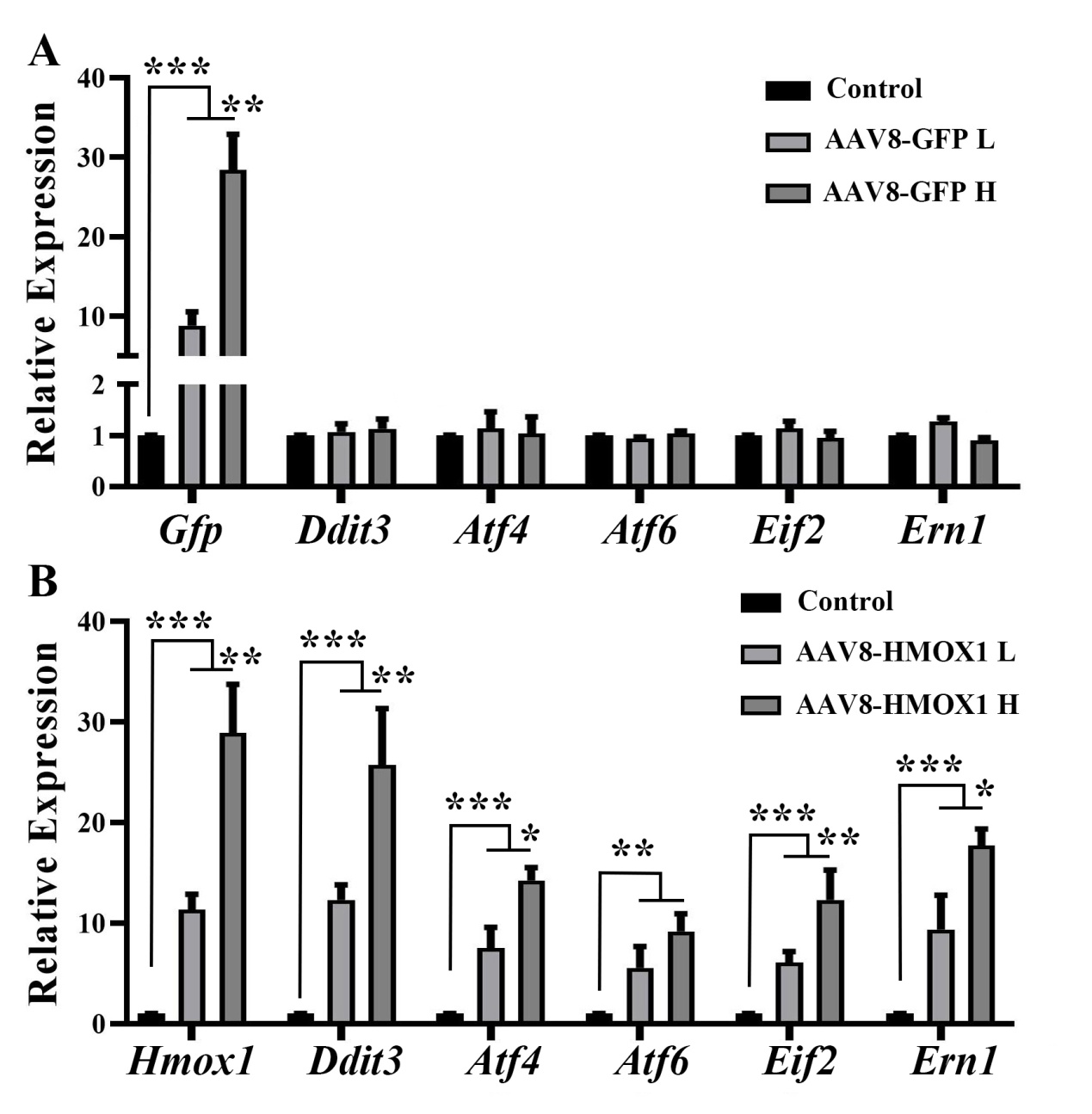


**Additional file 3:**

**Figure S3.** AAV8-HMOX1 up-regulates expressions of ER stress-related genes. 2-month-old albino mice were infected by the indicated virus and after two weeks subjected to gene expression analysis. **(A)** Quantification of q-RT-PCR shows expression of *Gfp* and other ER stress-related genes in the retinas infected with the low or the high dose of AAV8-GFP (Error bars: SD; n=3, one-way ANOVA). **(B)** Quantification of q-RT-PCR shows expression of *Hmox1* and ER stress-related genes in retinas infected by the low or the high dose of AAV8-HMOX1 after 2 weeks (Error bars: SD; n=3, one-way ANOVA). Note that upregulation of ER stress-related genes by AAV8-HMOX1 is dose-dependent. * or ** or *** indicates p<0.05 or p<0.01 or p<0.001.
