# Supplementary material for: High dose expression of heme oxigenase-1 induces retinal degeneration through ER stress-related DDIT3
Source: Mol Neurodegener. 2021 Mar 10;16:16. doi: 10.1186/s13024-021-00437-4 (PMC7944639; doi:10.1186/s13024-021-00437-4)
Supplement: Supplementary file 4 — Additional file 4 : Figure S4. AAV8-HMOX1 increases the level of non-heme iron in neural retinas. 2-month-old albino mice were infected with the indicated virus and after 2 weeks subjected to non-heme iron examination. (A, B) Images of chromogenic reaction solution from retinas infected with a low or high dose of AAV8-HMOX1 (A) or AAV8-GFP (B). “Reagent” corresponds to the reaction solution, and “Control” to the result obtained with neural retinas without virus infection. (C, D) Quantification of the relative level of non-heme iron in the retinas infected with the low or the high dose of AAV8-HMOX1 (C) or AAV8-GFP (D) (Error bars: SD; n = 5, one-way ANOVA). Note that increase of non-heme iron by AAV8-HMOX1 is dose-dependent. ** indicates p < 0.01. [file 13024_2021_437_MOESM4_ESM.docx]

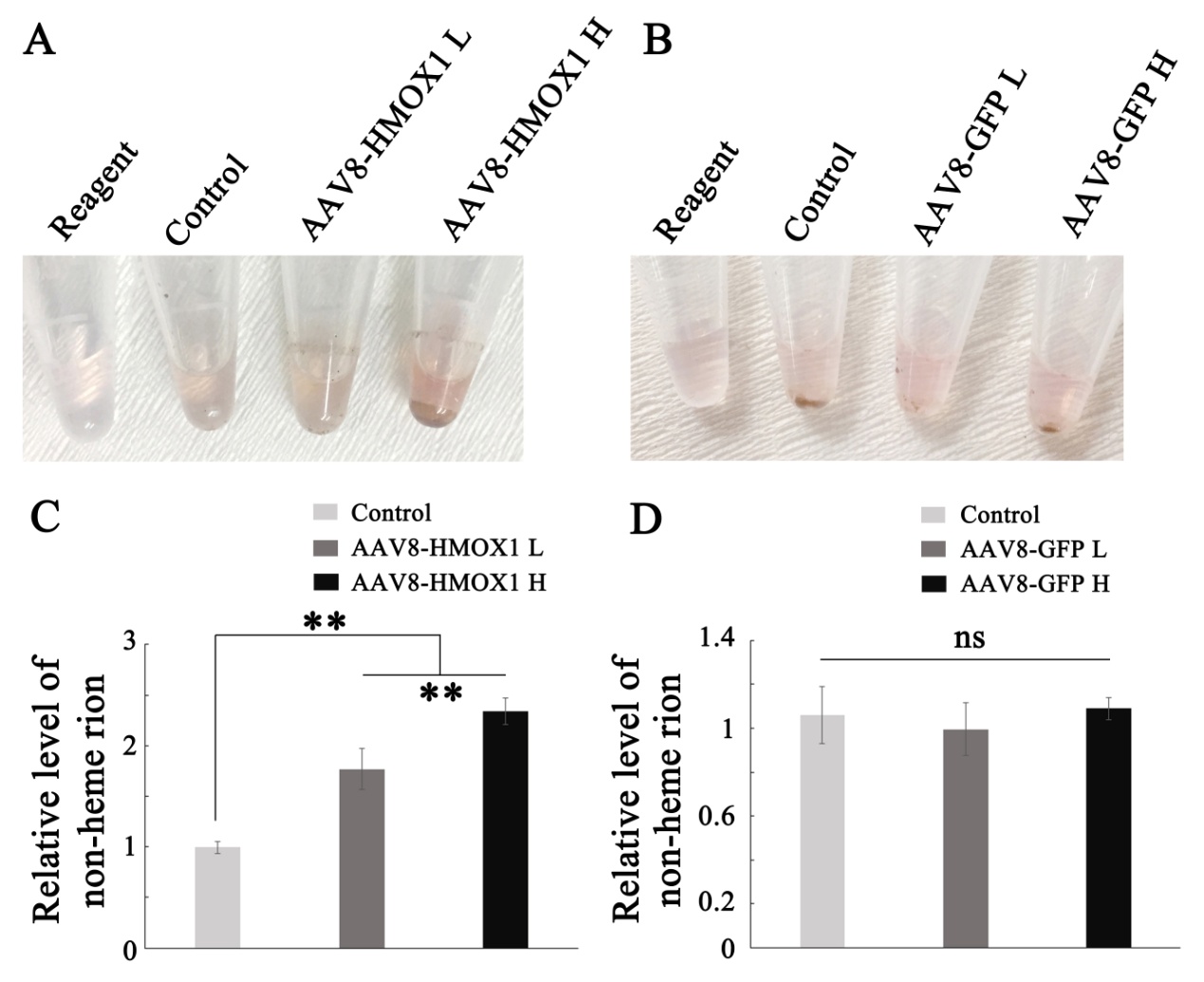


**Additional file 4:**

**Figure S4.** AAV8-HMOX1 increases the level of non-heme iron in neural retinas**.** 2-month-old albino mice were infected with the indicated virus and after two weeks subjected to non-heme iron examination. (**A, B**) Images of chromogenic reaction solution from retinas infected with a low or high dose of AAV8-HMOX1 (A) or AAV8-GFP (B). “Reagent” corresponds to the reaction solution, and “Control” to the result obtained with neural retinas without virus infection. (**C, D**) Quantification of the relative level of non-heme iron in the retinas infected with the low or the high dose of AAV8-HMOX1 (C) or AAV8-GFP (D) (Error bars: SD; n=5, one-way ANOVA). Note that increase of non-heme iron by AAV8-HMOX1 is dose-dependent. ** indicates p<0.01.
