# Supplementary material for: High dose expression of heme oxigenase-1 induces retinal degeneration through ER stress-related DDIT3
Source: Mol Neurodegener. 2021 Mar 10;16:16. doi: 10.1186/s13024-021-00437-4 (PMC7944639; doi:10.1186/s13024-021-00437-4)
Supplement: Supplementary file 5 — Additional file 5 : Figure S5. Iron-overload induces expressions of DDIT3 and ATF4 in photoreceptor cells. (A) 661 W photoreceptor cells were stimulated by the indicated doses of FAC (ferric ammonium citrate) and DDIT3 and ATF4 were determined using corresponding antibodies. (B) Quantification based on Western blot results shown in A (Error bars: SD; one-way ANOVA; n = 3; **, p < 0.01). Note that ER stress induced by FAC is dose-dependent in photoreceptor cells. [file 13024_2021_437_MOESM5_ESM.docx]

**
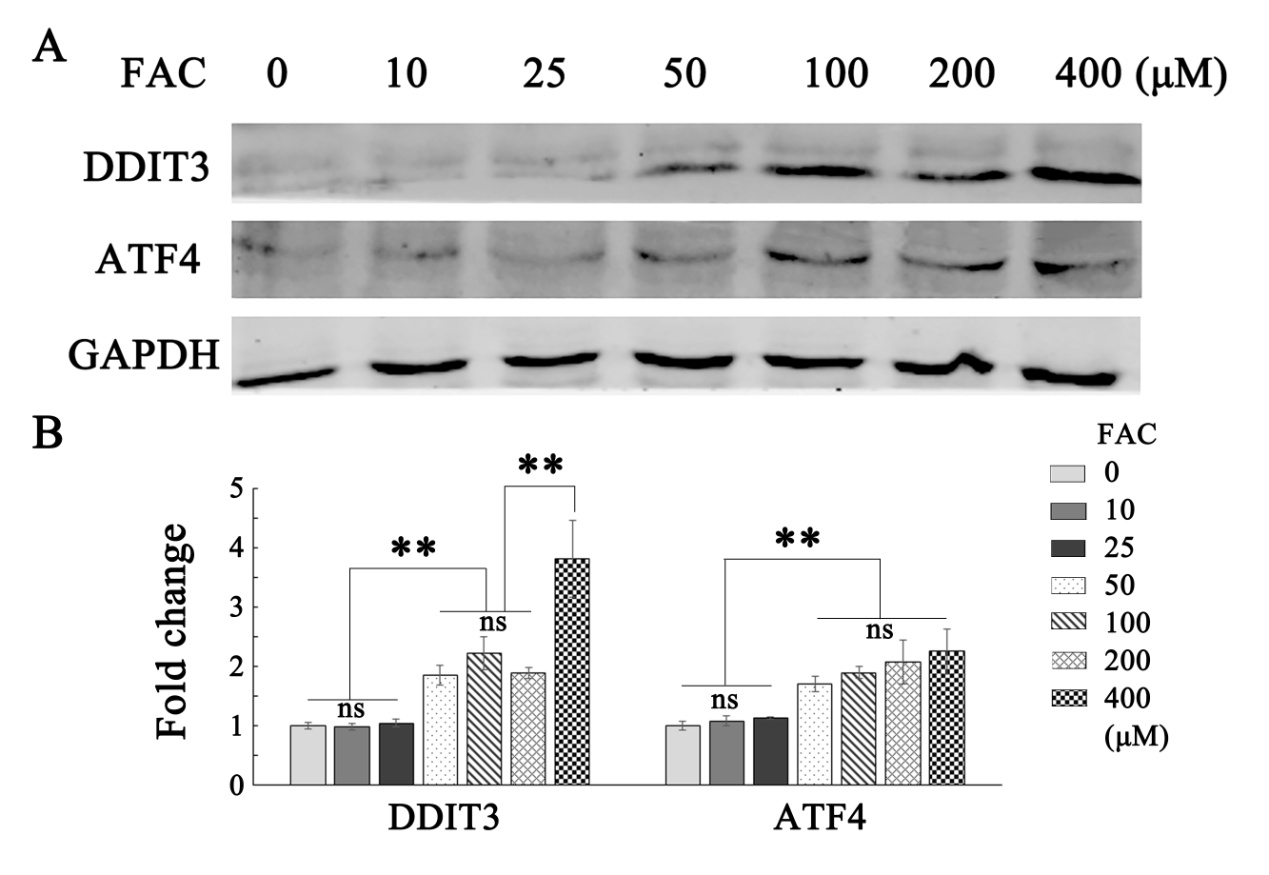
**

**Additional file 5:**

**Figure S5.** Iron-overload induces expressions of DDIT3 and ATF4 in photoreceptor cells**. (A)** 661W photoreceptor cells were stimulated by the indicated doses of FAC (ferric ammonium citrate) and DDIT3 and ATF4 were determined using corresponding antibodies. (**B)** Quantification based on Western blot results shown in A (Error bars: SD; one-way ANOVA; n=3; **, p<0.01). Note that ER stress induced by FAC is dose-dependent in photoreceptor cells.
