# Supplementary material for: High dose expression of heme oxigenase-1 induces retinal degeneration through ER stress-related DDIT3
Source: Mol Neurodegener. 2021 Mar 10;16:16. doi: 10.1186/s13024-021-00437-4 (PMC7944639; doi:10.1186/s13024-021-00437-4)
Supplement: Supplementary file 6 — Additional file 6 : Figure S6. Deletion of Ddit3 does not disrupt retinal function. (A) ERG traces of 2-month-old Ddit3+/+ (left panels) and Ddit3−/− mice (right panels) in rod response and standard response. (B, C) Quantification of ERG amplitudes in rod response (B) and standard response (C) according to the results form B (Error bars: SD; n = 4, Student T-test). [file 13024_2021_437_MOESM6_ESM.docx]

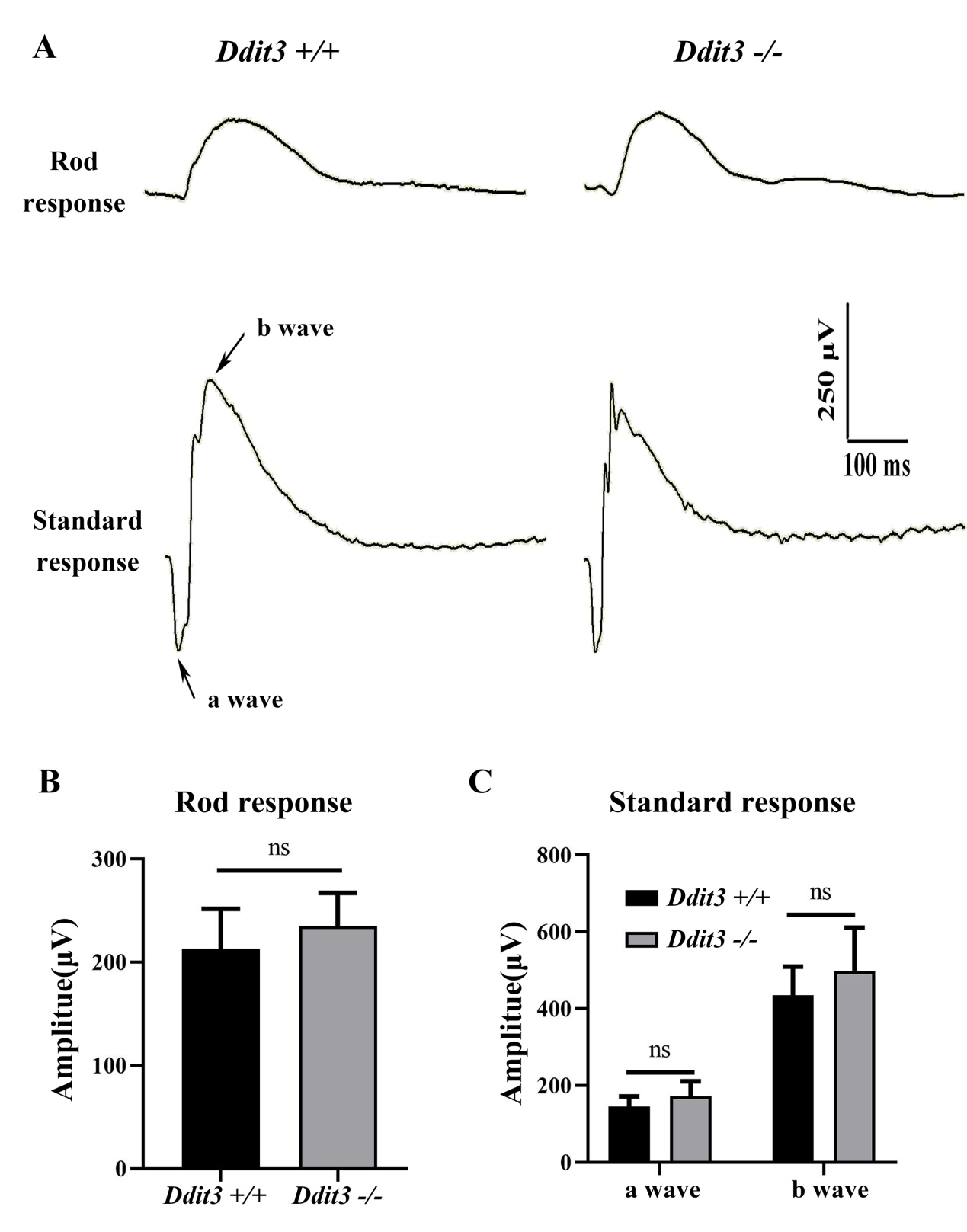


**Additional file 6:**

**Figure S6.** Deletion of *Ddit3* does not disrupt retinal function**. (A)** ERG traces of 2-month-old *Ddit3+/+* (left panels) and *Ddit3-/-* mice (right panels) in rod response and standard response**. (B, C)** Quantification of ERG amplitudes in rod response (**B**) and standard response (**C**) according to the results form B (Error bars: SD; n=4, Student *T*-test).
