# Supplementary material for: High dose expression of heme oxigenase-1 induces retinal degeneration through ER stress-related DDIT3
Source: Mol Neurodegener. 2021 Mar 10;16:16. doi: 10.1186/s13024-021-00437-4 (PMC7944639; doi:10.1186/s13024-021-00437-4)
Supplement: Supplementary file 7 — Additional file 7 : Figure S7. Deletion of Ddit3 prevents photoreceptor cell death induced by the AAV8-mediated high dose of HMOX1. (A) Representative images of TUNEL assays from 2-month-old Ddit3+/+ (left panels) and Ddit3−/− (right panels) retinas 2 weeks after infection with the high dose of AAV8-GFP (upper panels) or of AAV8-HMOX1 (lower panels). (B) Quantification of cell death in the ONL of the Ddit3+/+ and Ddit3−/− retinas infected with the high dose of AAV8-GFP or of AAV8-HMOX1 (Error bars: SD; n = 4, one-way ANOVA). Note that deletion of Ddit3 rescues photoreceptor cell death caused by the high dose of AAV8-HMOX1. INL, inner nuclear layer; ONL, outer nuclear layer. ** indicates p < 0.01. Scale bar: 50 μm. [file 13024_2021_437_MOESM7_ESM.docx]

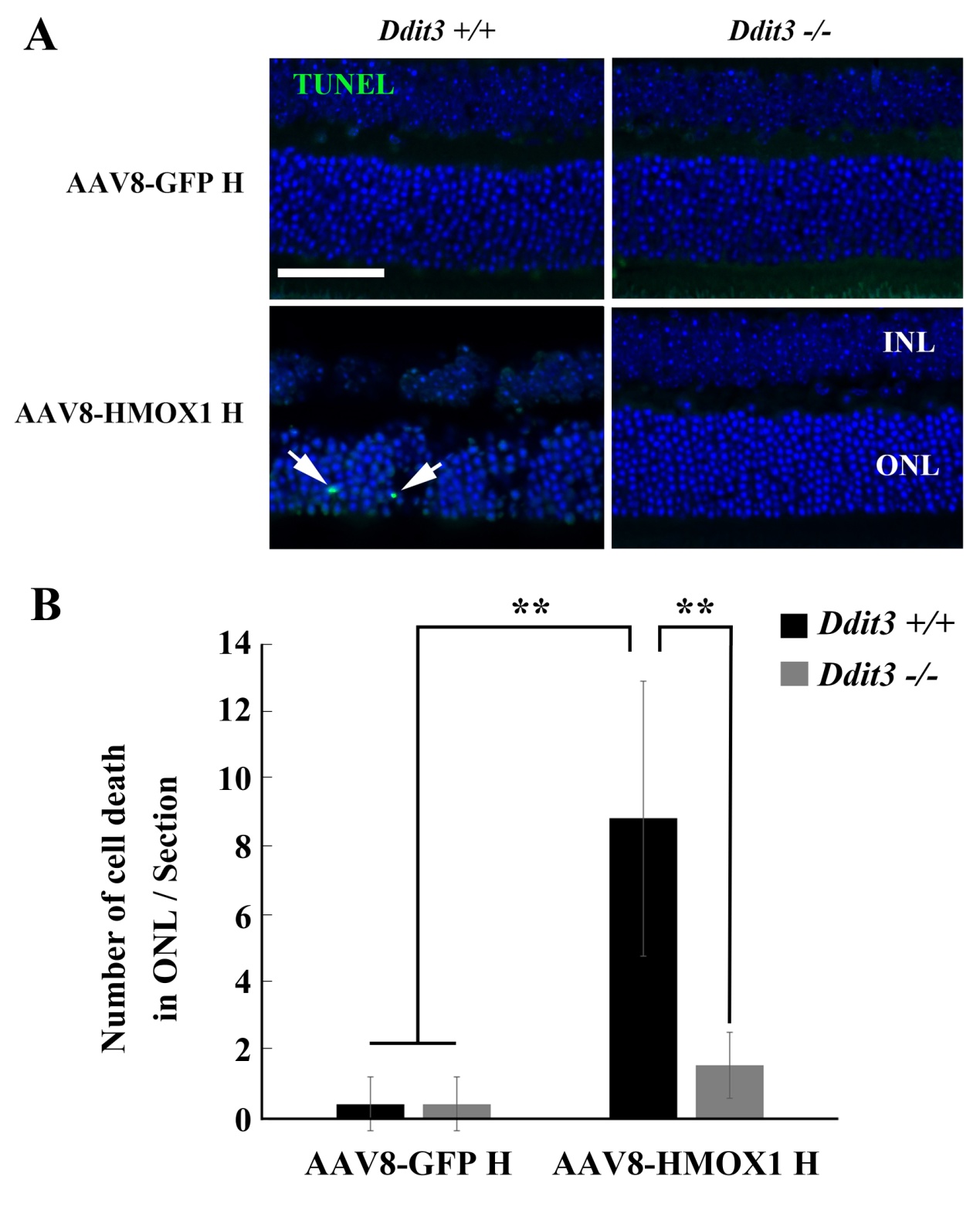


**Additional file 7:**

**Figure S7.** Deletion of *Ddit3* prevents photoreceptor cell death induced by the AAV8-mediated high dose of HMOX1**.** (**A**) Representative images of TUNEL assays from 2-month-old *Ddit3+/+* (left panels) and *Ddit3-/-* (right panels) retinas 2 weeks after infection with the high dose of AAV8-GFP (upper panels) or of AAV8-HMOX1 (lower panels). (**B**) Quantification of cell death in the ONL of the *Ddit3+/+* and *Ddit3-/-* retinas infected with the high dose of AAV8-GFP or of AAV8-HMOX1 (Error bars: SD; n=4, one-way ANOVA). Note that deletion of *Ddit3* rescues photoreceptor cell death caused by the high dose of AAV8-HMOX1**.** INL, inner nuclear layer; ONL, outer nuclear layer. ** indicates p<0.01. Scale bar: 50 μm.
