# Supplementary material for: High dose expression of heme oxigenase-1 induces retinal degeneration through ER stress-related DDIT3
Source: Mol Neurodegener. 2021 Mar 10;16:16. doi: 10.1186/s13024-021-00437-4 (PMC7944639; doi:10.1186/s13024-021-00437-4)
Supplement: Supplementary file 8 — Additional file 8 : Table 1. Sequences of primers used for gene expression. [file 13024_2021_437_MOESM8_ESM.docx]

**Additional Table 1. Sequences of primers used for gene expression**

| Gene name | NCBI Gene ID | Forward primer | Reverse primer |
| --- | --- | --- | --- |
| Gapdh | 14433 | ACCACAGTCCATGCCATCAC | TCCACCACCCTGTTGCTGTA |
| Rho | 212541 | CCCATCAACTTCCTCACGCT | AGGGCGATTTCACCTCCAAG |
| Gfap | 14580 | AGGGCGAAGAAAACCGCATC | GGTGAGCCTGTATTGGGACA |
| Ddit3 | 13198 | CACCTGAAAGCAGAACCTGG | GGACGCAGGGTCAAGAGTAG |
| Eif2 | 67204 | CCGCAAGAAGGACCCTATCC | CAGACTCCTTCCGCTGCCTG |
| Atf4 | 11911 | CGCTGCGGTAGGATCACG | TGAAGAGCGCCATGGCTTAG |
| Atf6 | 226641 | ACTCACCCATCCGAGTTGTG | ACAACGTCGACTCCCAGTCT |
| Hmox1 | 15368 | CAGAAGAGGCTAAGACCGCC | GCAGTATCTTGCACCAGGCTA |
| Ern1 | 78943 | ACGAAGGCCTGACGAAACTT | ATCAGCAAAGGCCGATGACA |
